# Supplementary material for: Enhancing Transcriptomic Insights into Neurological Disorders Through the Comparative Analysis of Shapley Values
Source: Curr Issues Mol Biol. 2024 Nov 29;46(12):13583–606. doi: 10.3390/cimb46120812 (PMC11726880; doi:10.3390/cimb46120812)
Supplement: Supplementary file 1 [file cimb-46-00812-s001.zip › SupplementaryFigure_S1.pptx]

## Slide 1
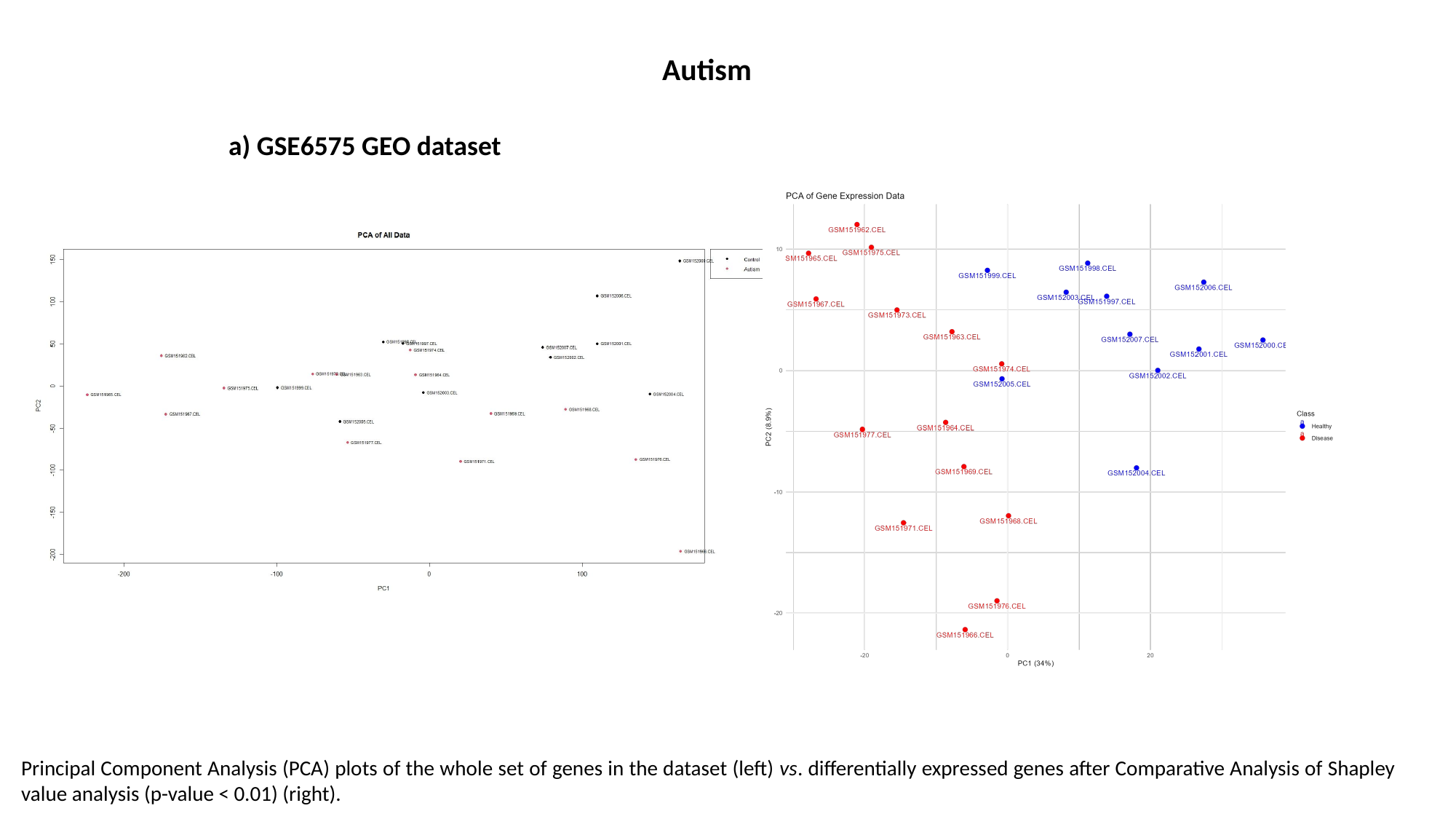

Autism
a) GSE6575 GEO dataset
Principal Component Analysis (PCA) plots of the whole set of genes in the dataset (left) vs. differentially expressed genes after Comparative Analysis of Shapley value analysis (p-value < 0.01) (right).

## Slide 2
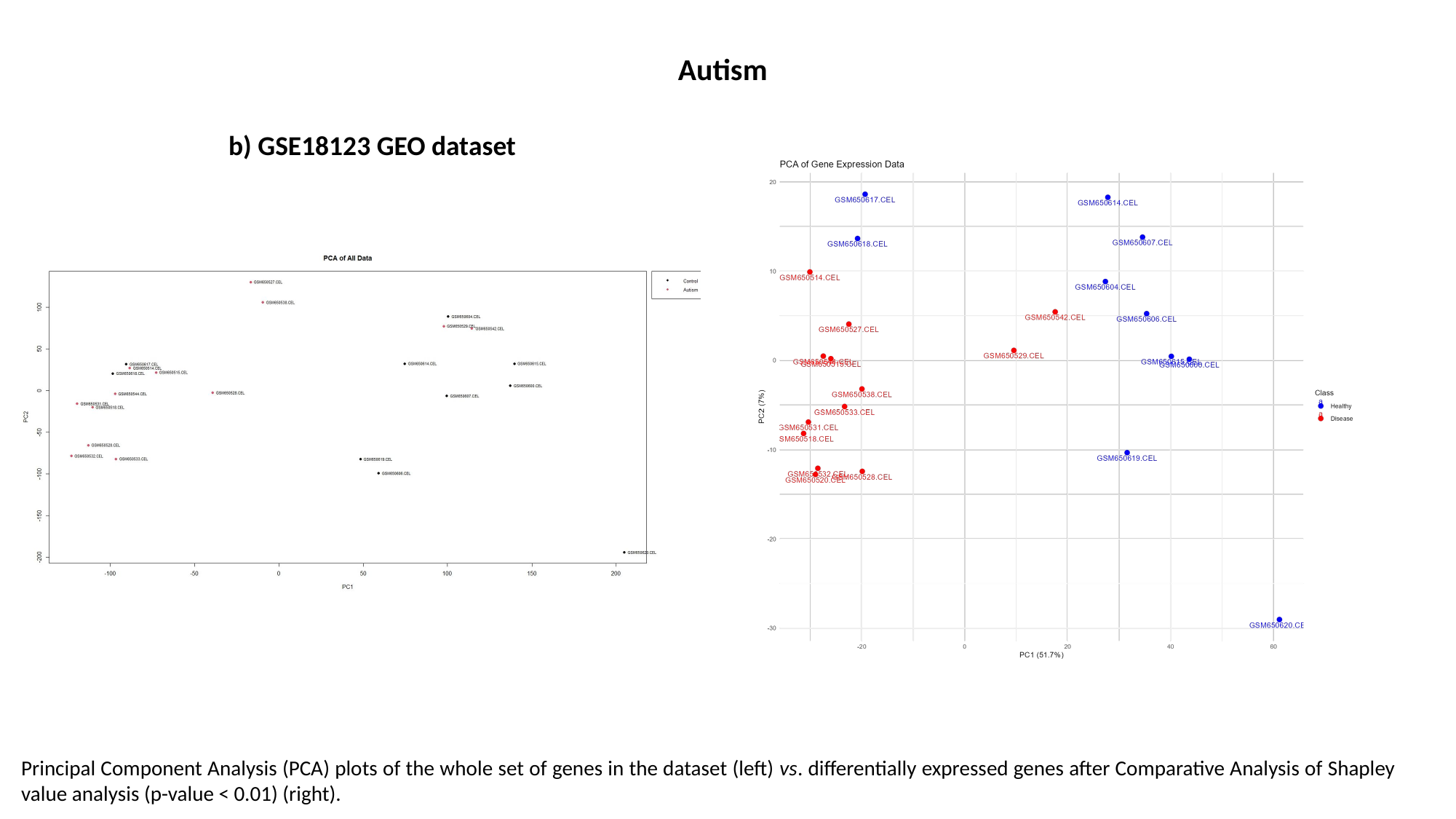

Autism
b) GSE18123 GEO dataset
Principal Component Analysis (PCA) plots of the whole set of genes in the dataset (left) vs. differentially expressed genes after Comparative Analysis of Shapley value analysis (p-value < 0.01) (right).

## Slide 3
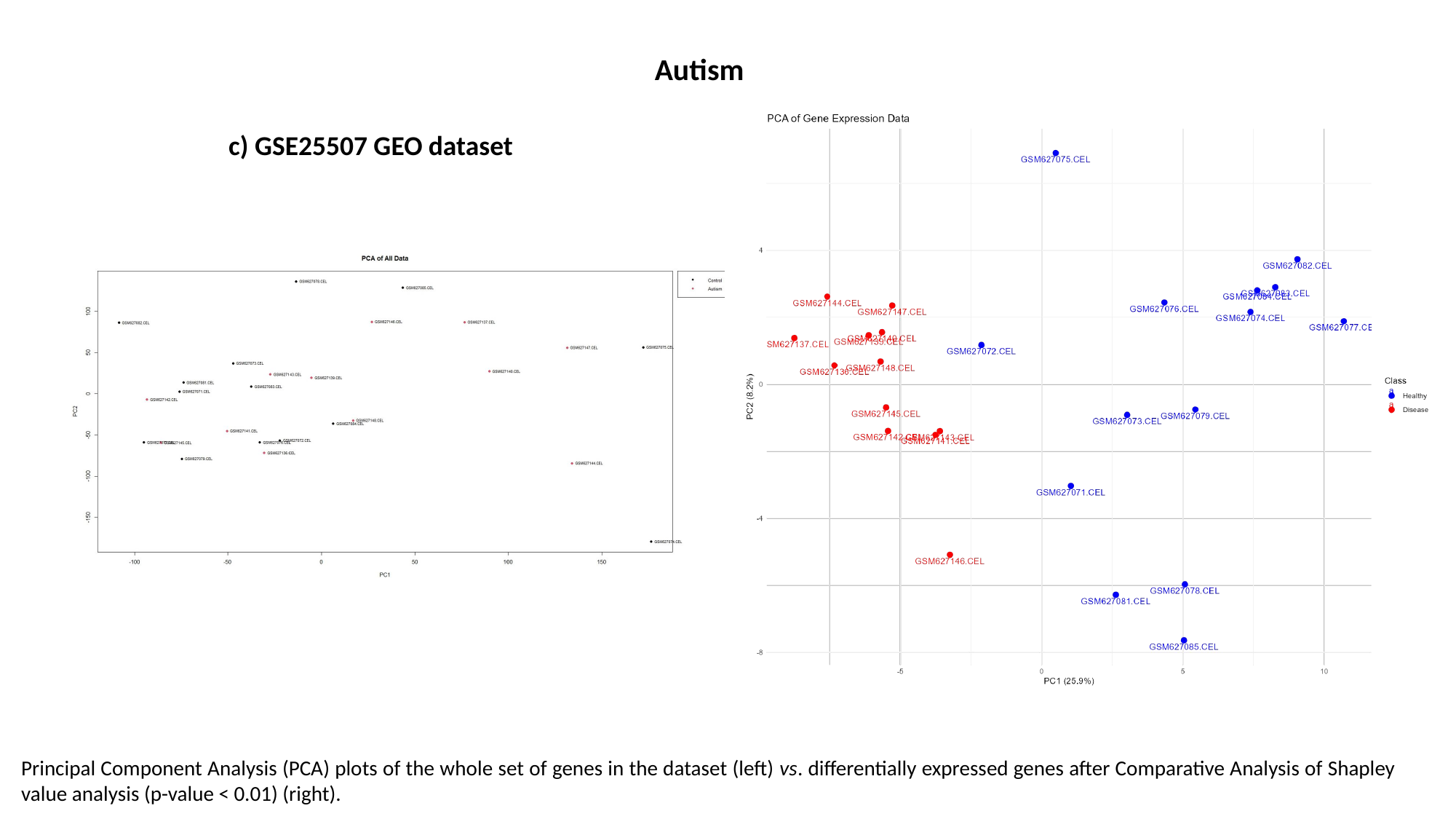

Autism
c) GSE25507 GEO dataset
Principal Component Analysis (PCA) plots of the whole set of genes in the dataset (left) vs. differentially expressed genes after Comparative Analysis of Shapley value analysis (p-value < 0.01) (right).

## Slide 4
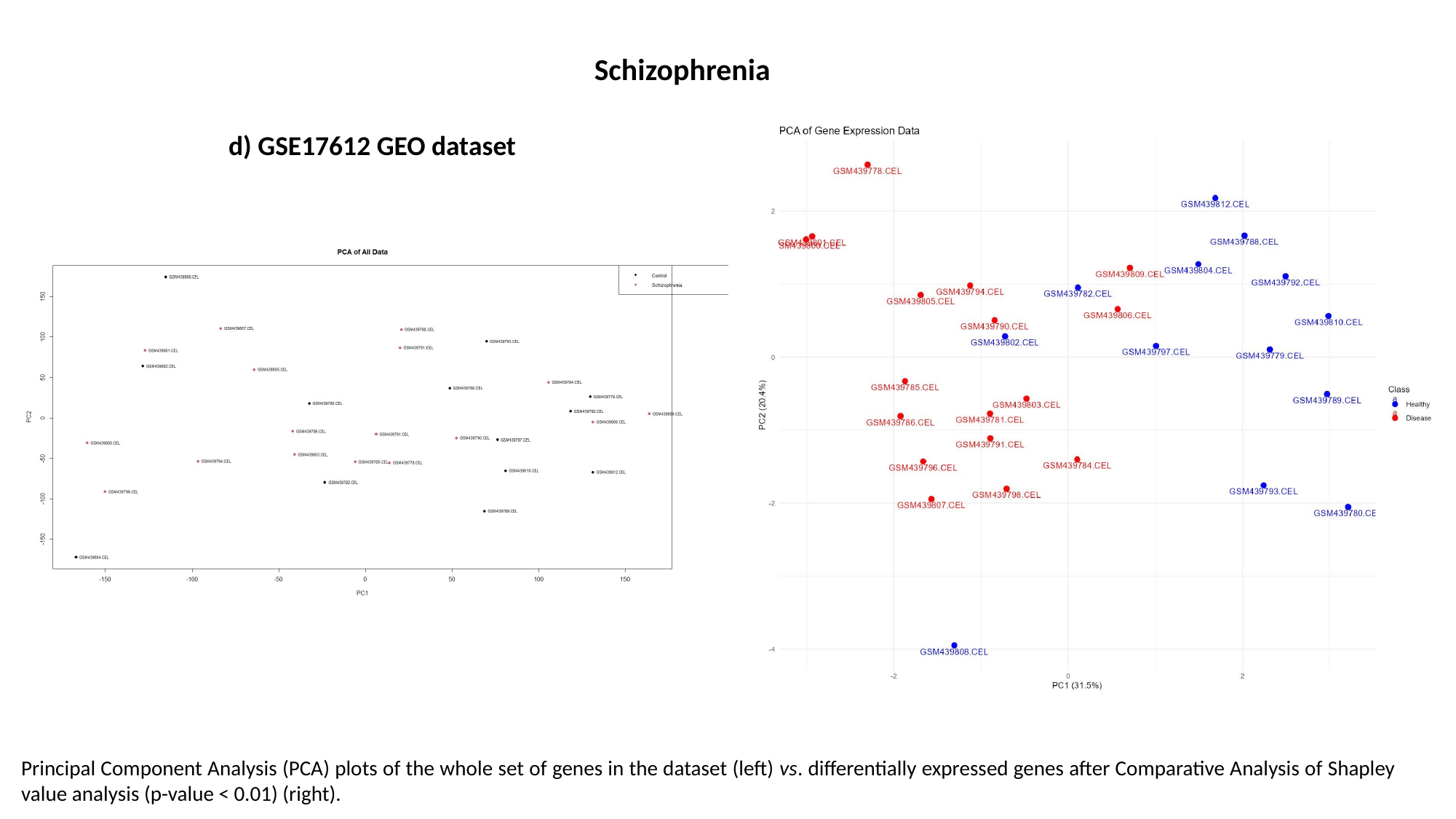

Schizophrenia
d) GSE17612 GEO dataset
Principal Component Analysis (PCA) plots of the whole set of genes in the dataset (left) vs. differentially expressed genes after Comparative Analysis of Shapley value analysis (p-value < 0.01) (right).

## Slide 5
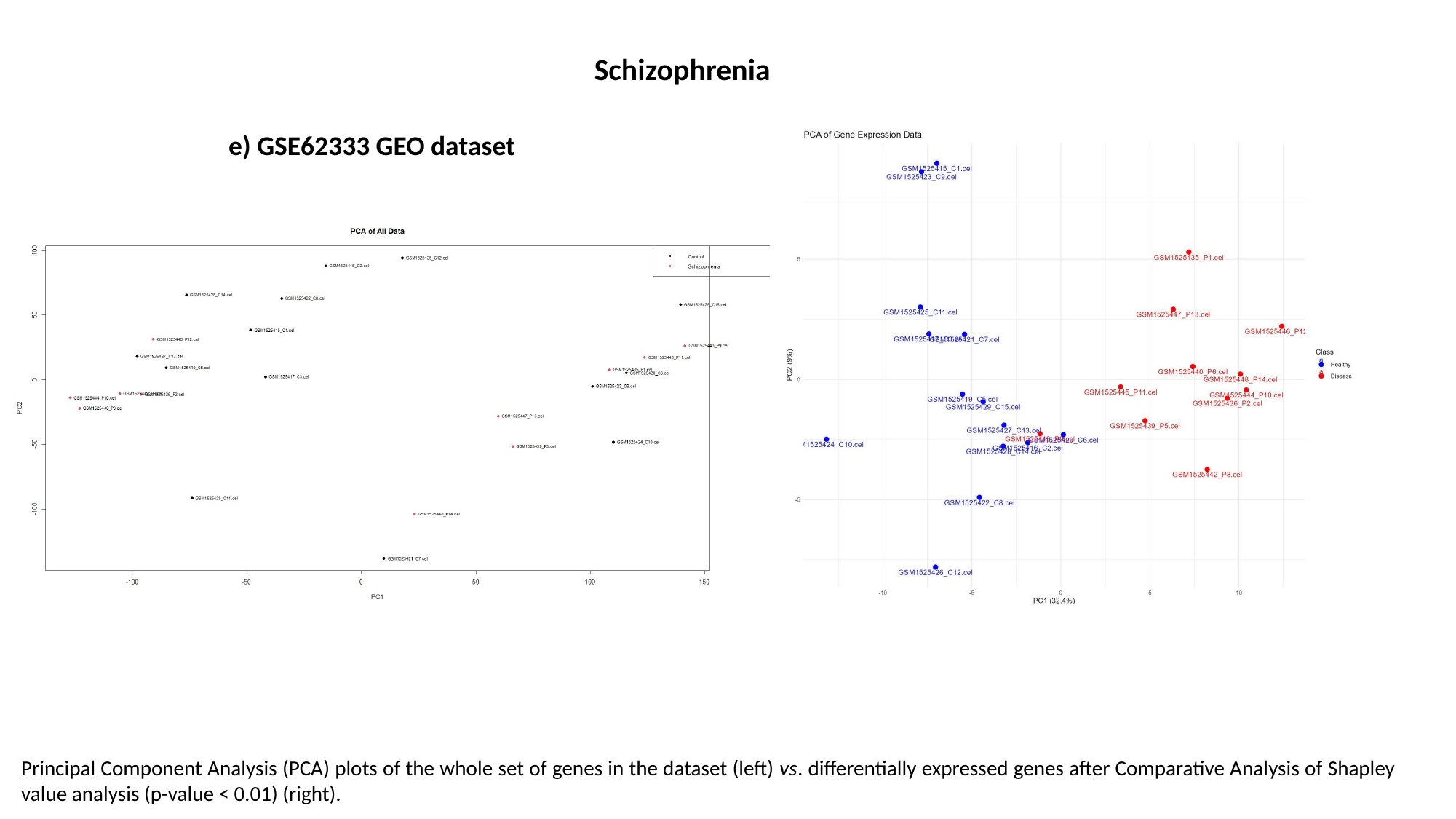

Schizophrenia
e) GSE62333 GEO dataset
Principal Component Analysis (PCA) plots of the whole set of genes in the dataset (left) vs. differentially expressed genes after Comparative Analysis of Shapley value analysis (p-value < 0.01) (right).

## Slide 6
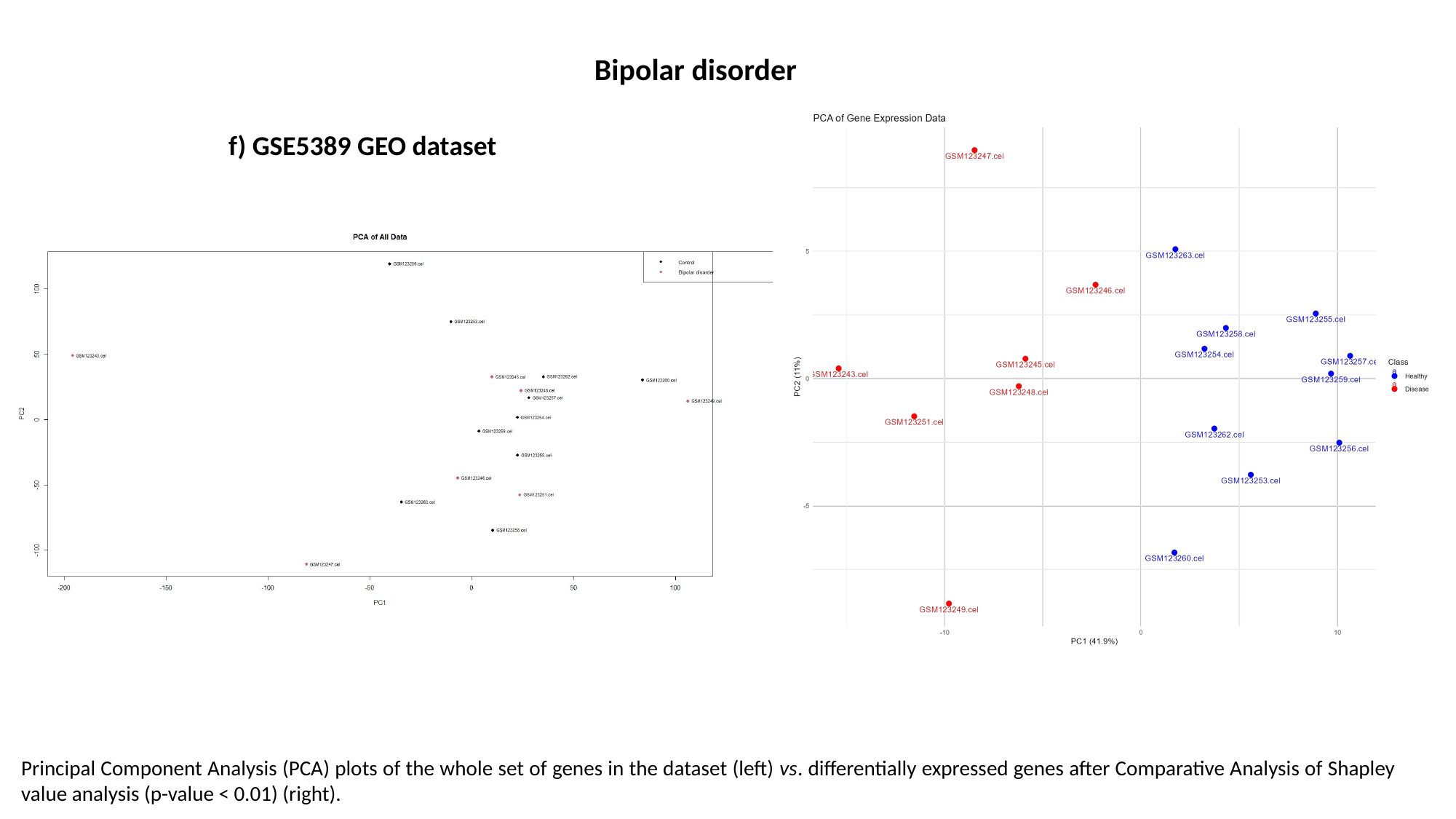

Bipolar disorder
f) GSE5389 GEO dataset
Principal Component Analysis (PCA) plots of the whole set of genes in the dataset (left) vs. differentially expressed genes after Comparative Analysis of Shapley value analysis (p-value < 0.01) (right).

## Slide 7
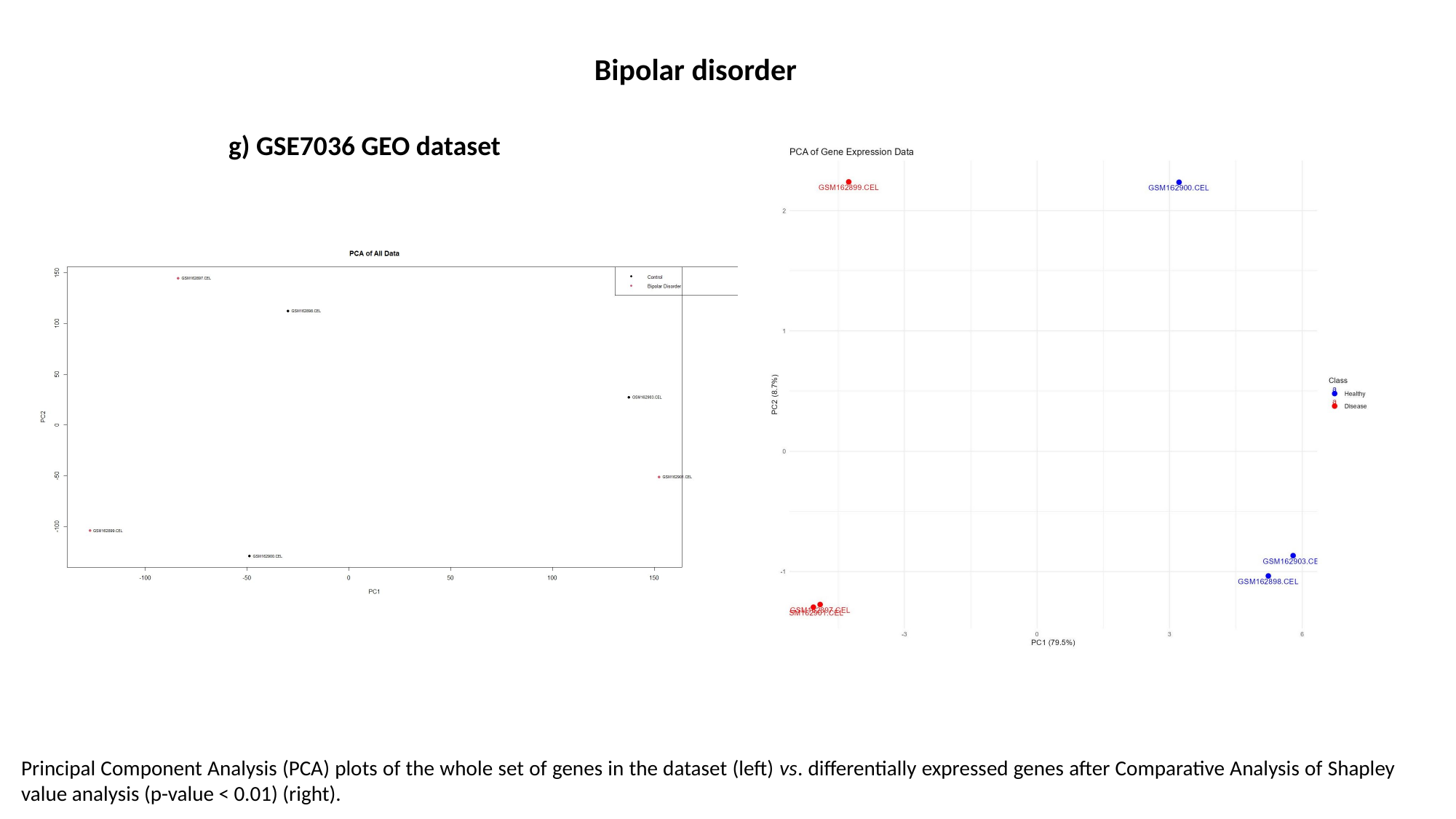

Bipolar disorder
g) GSE7036 GEO dataset
Principal Component Analysis (PCA) plots of the whole set of genes in the dataset (left) vs. differentially expressed genes after Comparative Analysis of Shapley value analysis (p-value < 0.01) (right).

## Slide 8
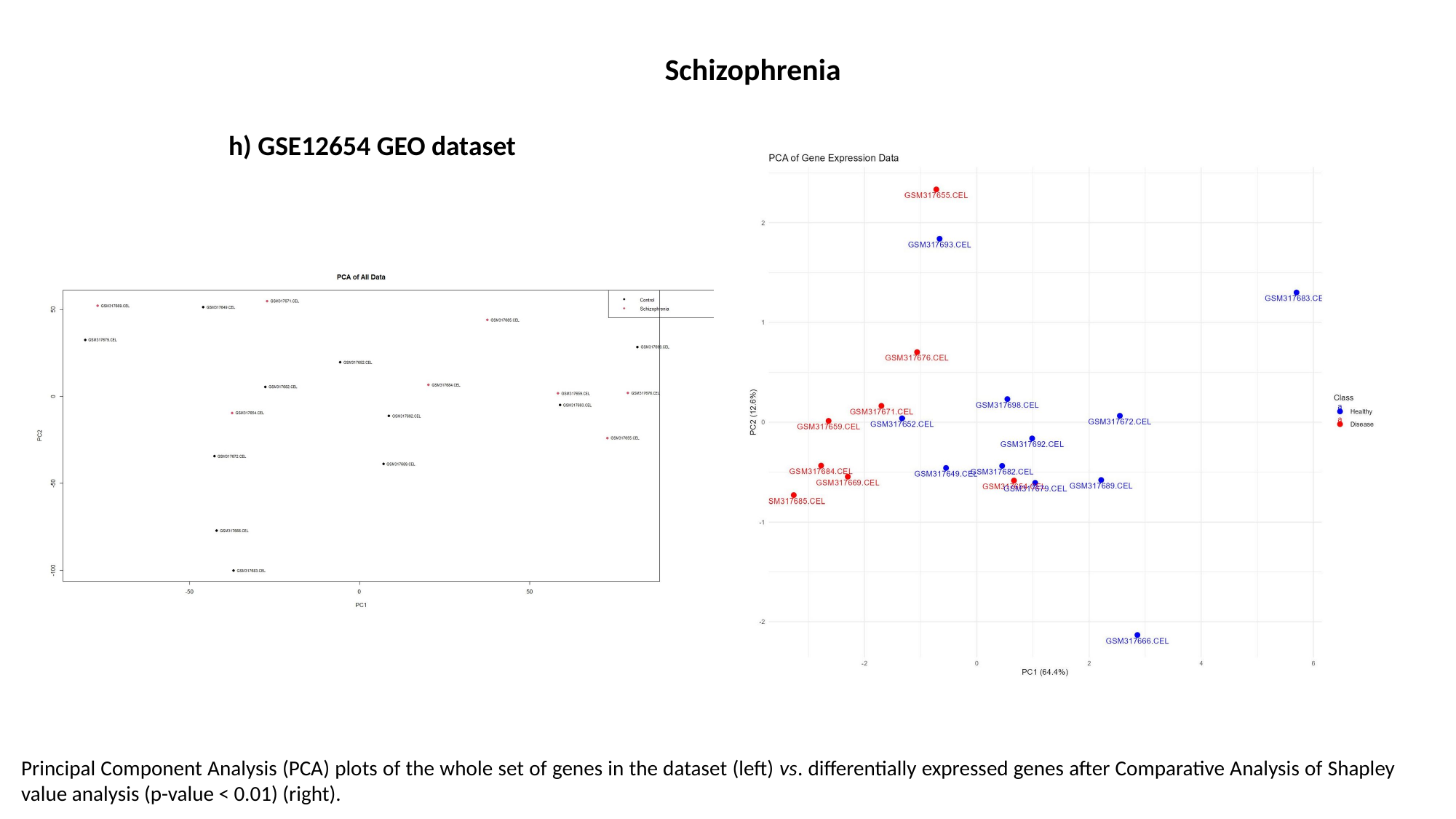

Schizophrenia
h) GSE12654 GEO dataset
Principal Component Analysis (PCA) plots of the whole set of genes in the dataset (left) vs. differentially expressed genes after Comparative Analysis of Shapley value analysis (p-value < 0.01) (right).

## Slide 9
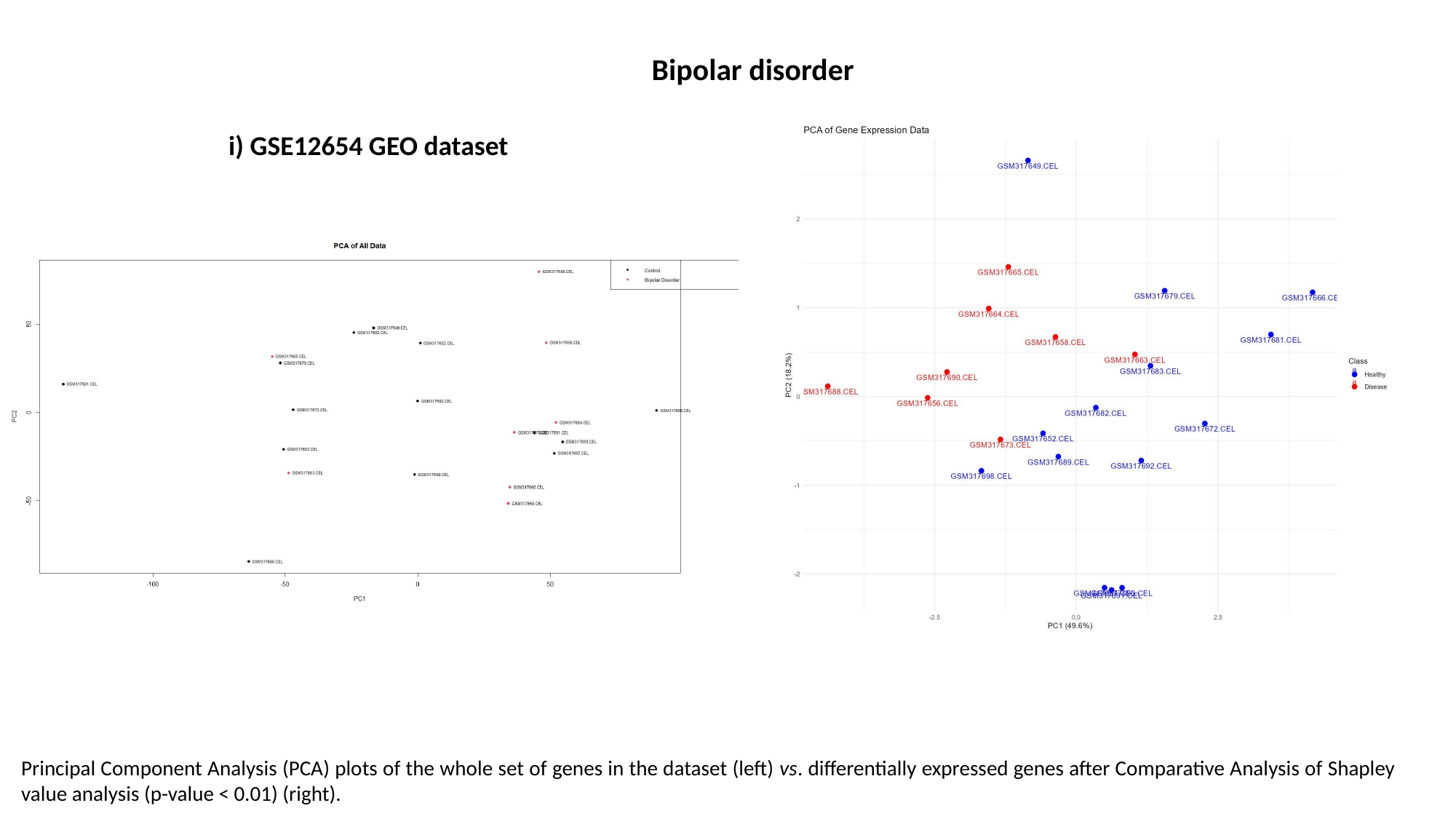

Bipolar disorder
i) GSE12654 GEO dataset
Principal Component Analysis (PCA) plots of the whole set of genes in the dataset (left) vs. differentially expressed genes after Comparative Analysis of Shapley value analysis (p-value < 0.01) (right).

## Slide 10
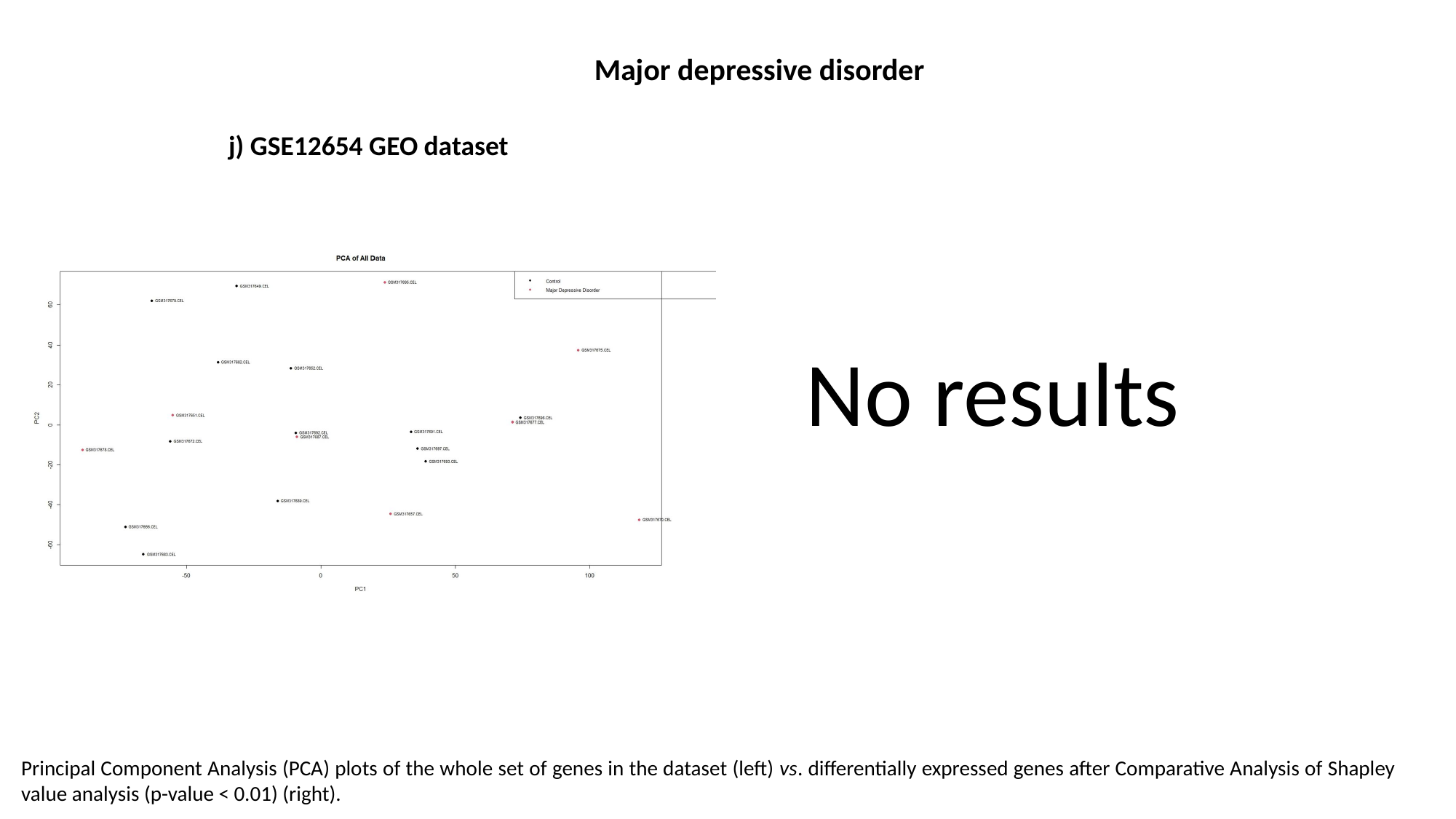

Major depressive disorder
j) GSE12654 GEO dataset
No results
Principal Component Analysis (PCA) plots of the whole set of genes in the dataset (left) vs. differentially expressed genes after Comparative Analysis of Shapley value analysis (p-value < 0.01) (right).

## Slide 11
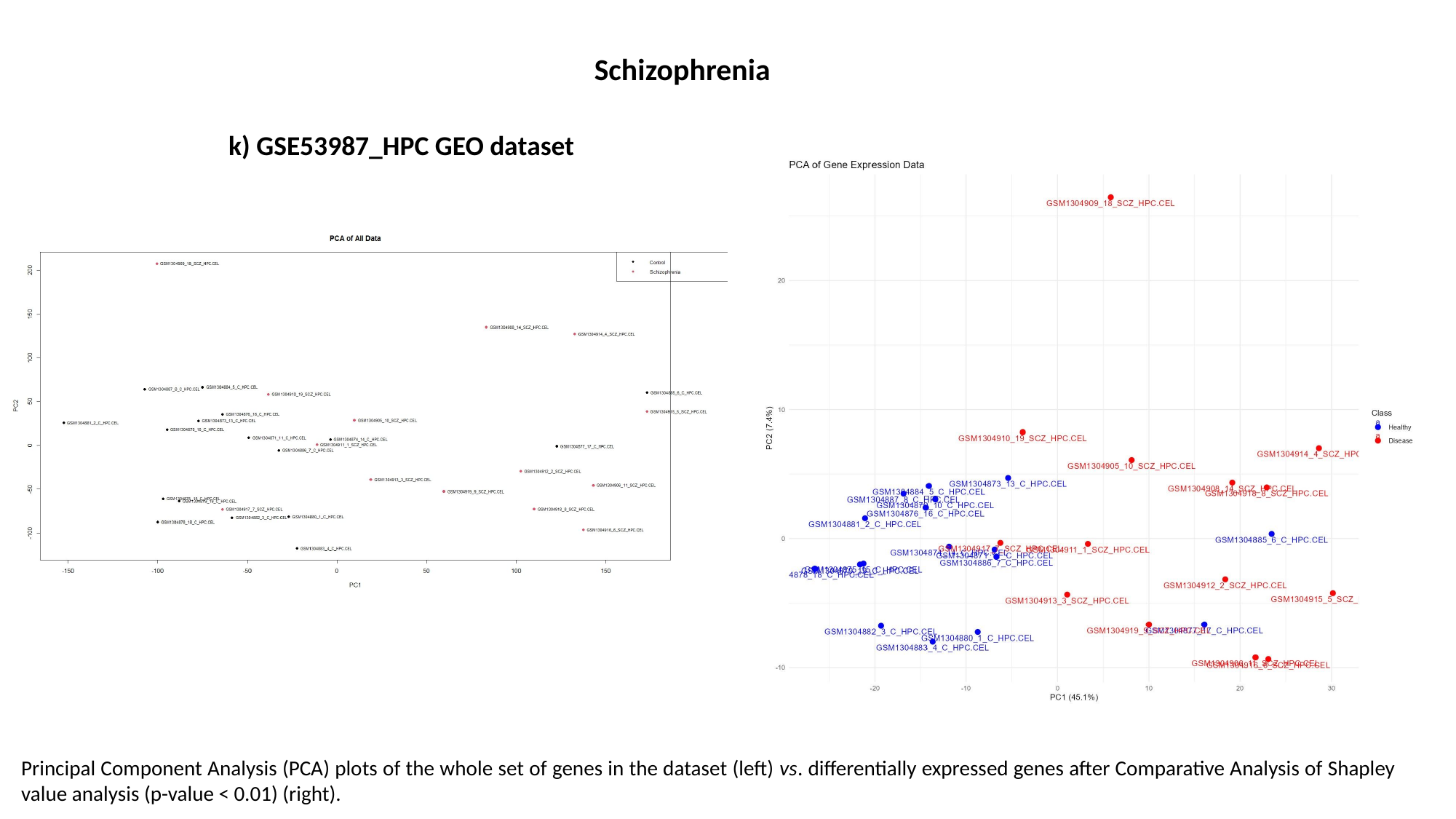

Schizophrenia
k) GSE53987_HPC GEO dataset
Principal Component Analysis (PCA) plots of the whole set of genes in the dataset (left) vs. differentially expressed genes after Comparative Analysis of Shapley value analysis (p-value < 0.01) (right).

## Slide 12
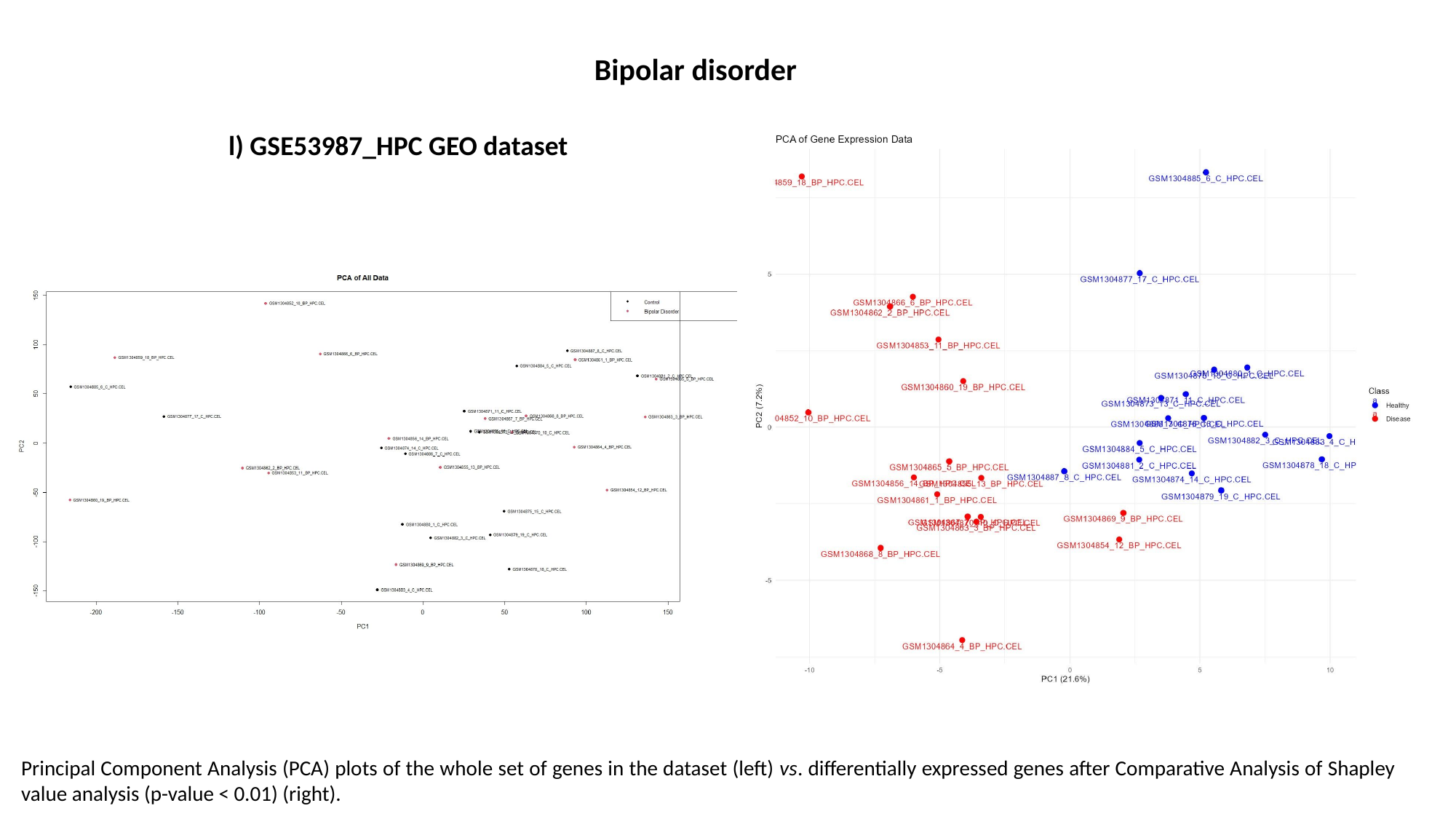

Bipolar disorder
l) GSE53987_HPC GEO dataset
Principal Component Analysis (PCA) plots of the whole set of genes in the dataset (left) vs. differentially expressed genes after Comparative Analysis of Shapley value analysis (p-value < 0.01) (right).

## Slide 13
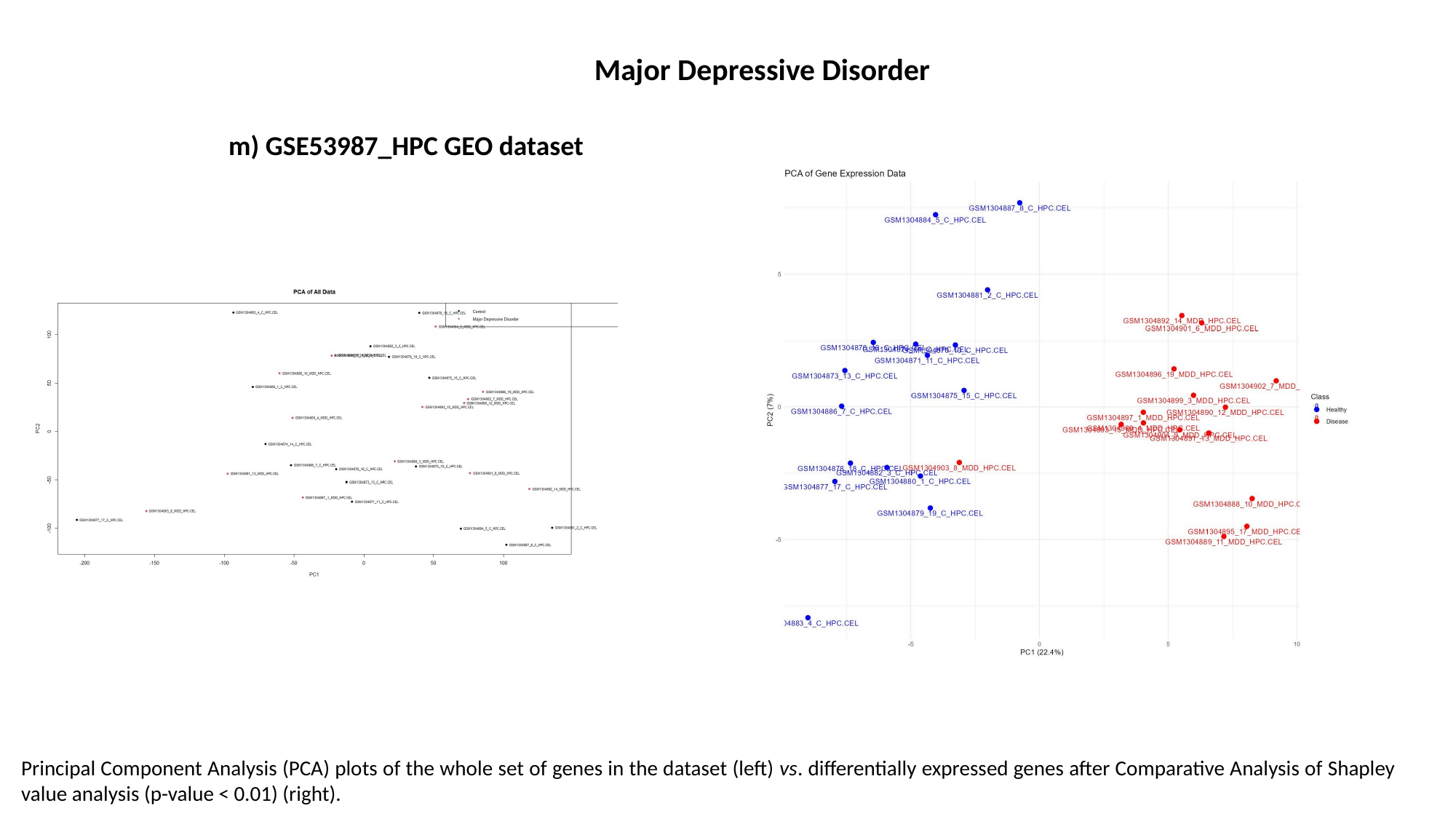

Major Depressive Disorder
m) GSE53987_HPC GEO dataset
Principal Component Analysis (PCA) plots of the whole set of genes in the dataset (left) vs. differentially expressed genes after Comparative Analysis of Shapley value analysis (p-value < 0.01) (right).

## Slide 14
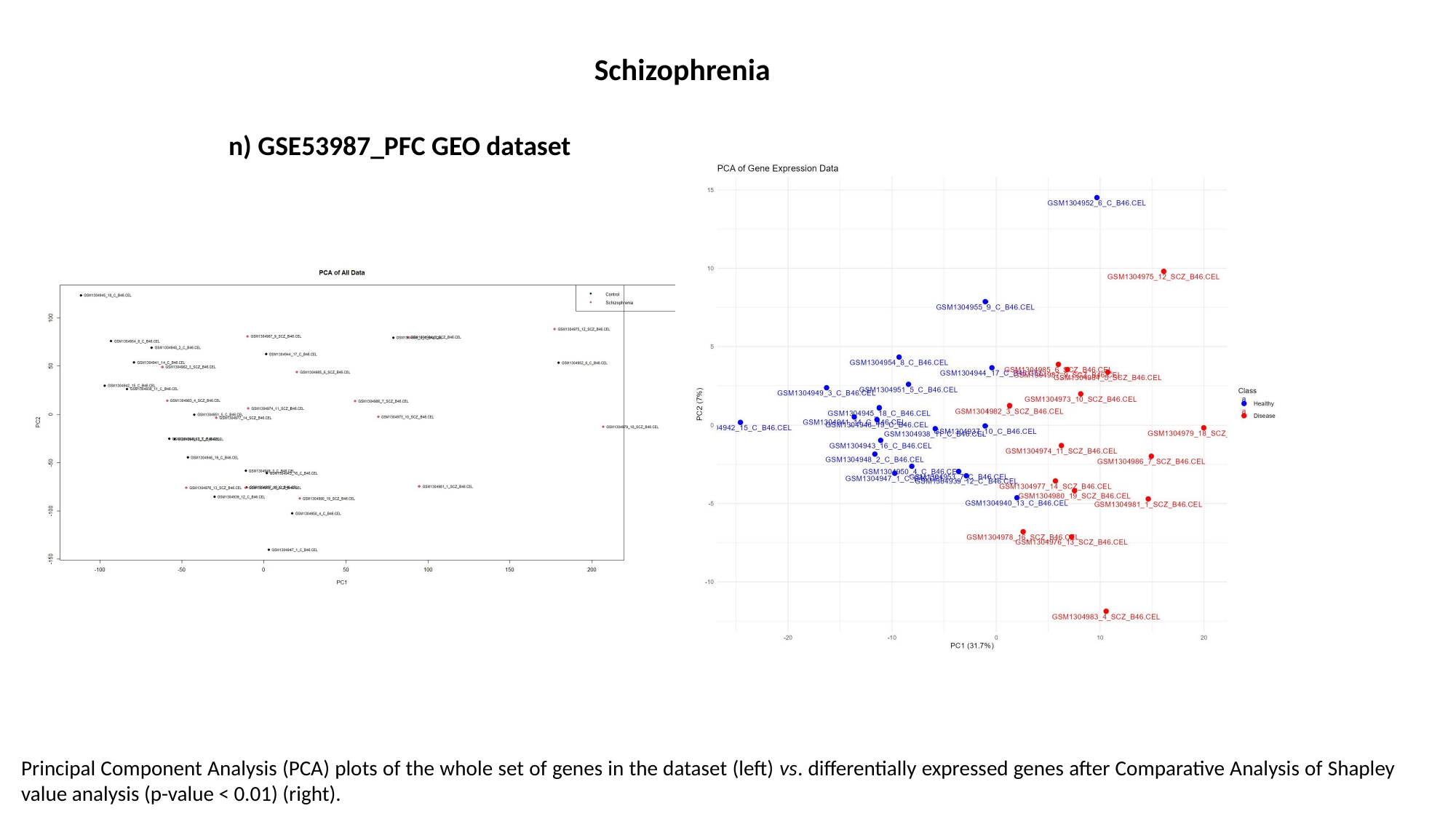

Schizophrenia
n) GSE53987_PFC GEO dataset
Principal Component Analysis (PCA) plots of the whole set of genes in the dataset (left) vs. differentially expressed genes after Comparative Analysis of Shapley value analysis (p-value < 0.01) (right).

## Slide 15
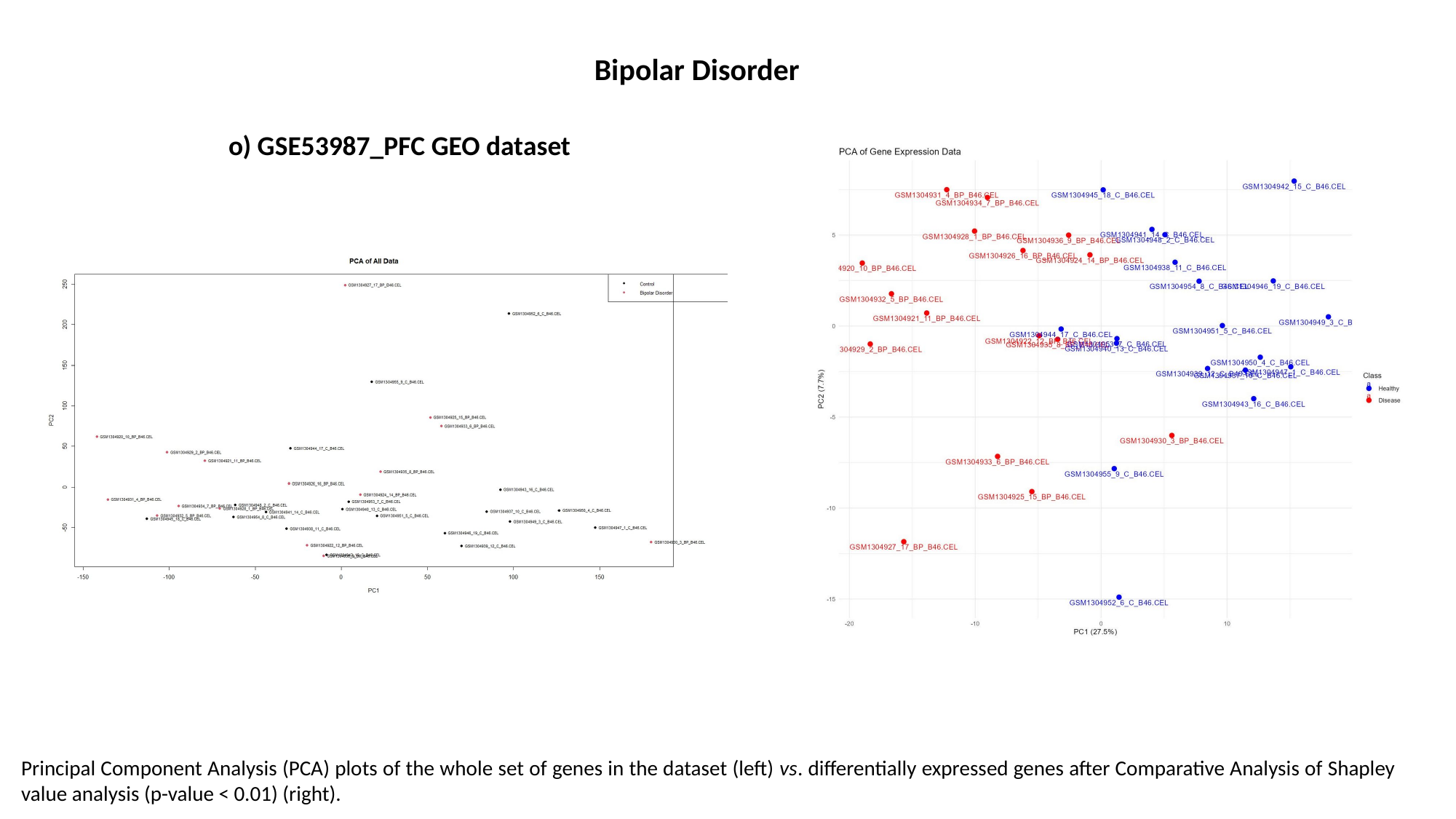

Bipolar Disorder
o) GSE53987_PFC GEO dataset
Principal Component Analysis (PCA) plots of the whole set of genes in the dataset (left) vs. differentially expressed genes after Comparative Analysis of Shapley value analysis (p-value < 0.01) (right).

## Slide 16
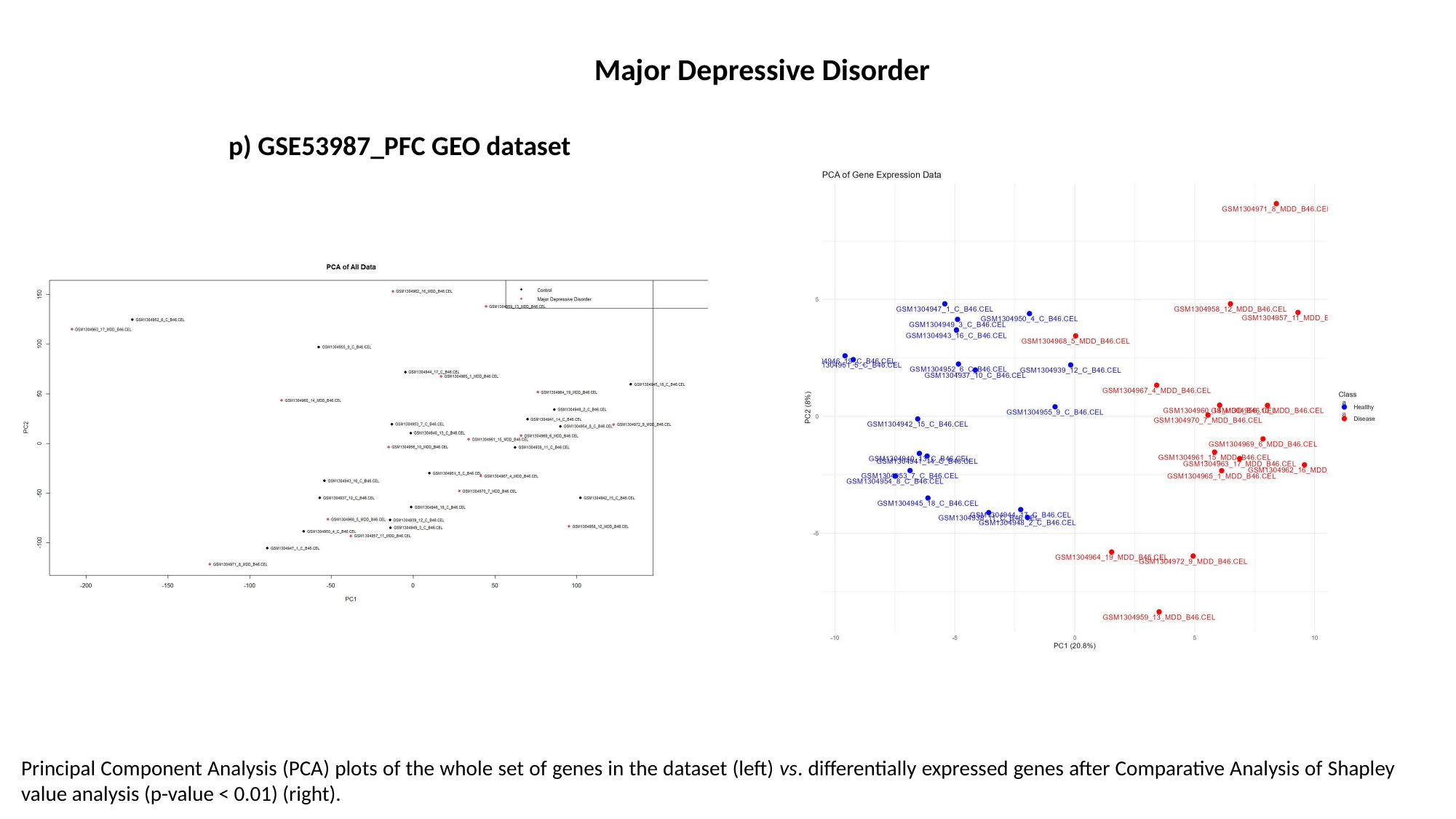

Major Depressive Disorder
p) GSE53987_PFC GEO dataset
Principal Component Analysis (PCA) plots of the whole set of genes in the dataset (left) vs. differentially expressed genes after Comparative Analysis of Shapley value analysis (p-value < 0.01) (right).

## Slide 17
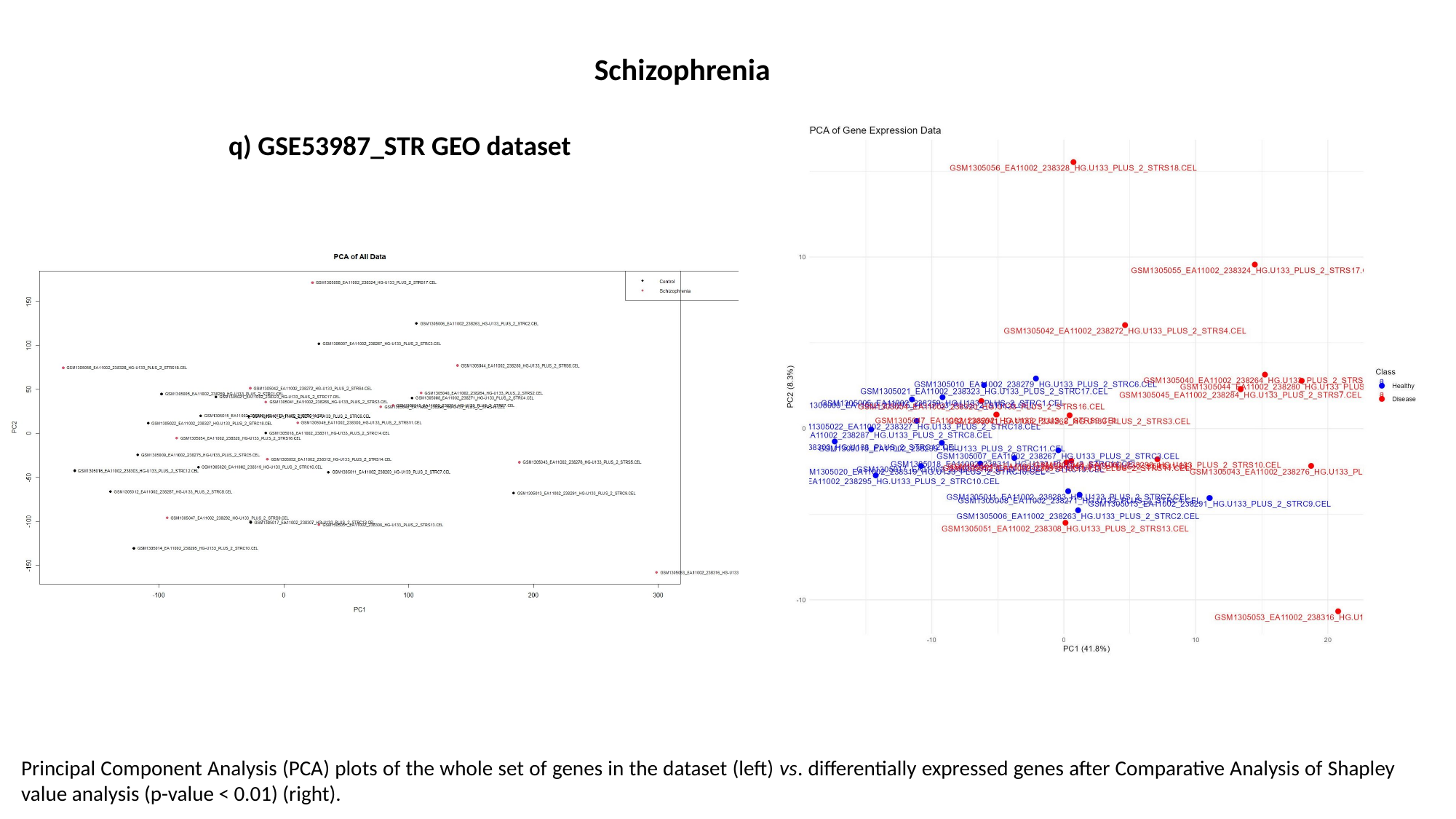

Schizophrenia
q) GSE53987_STR GEO dataset
Principal Component Analysis (PCA) plots of the whole set of genes in the dataset (left) vs. differentially expressed genes after Comparative Analysis of Shapley value analysis (p-value < 0.01) (right).

## Slide 18
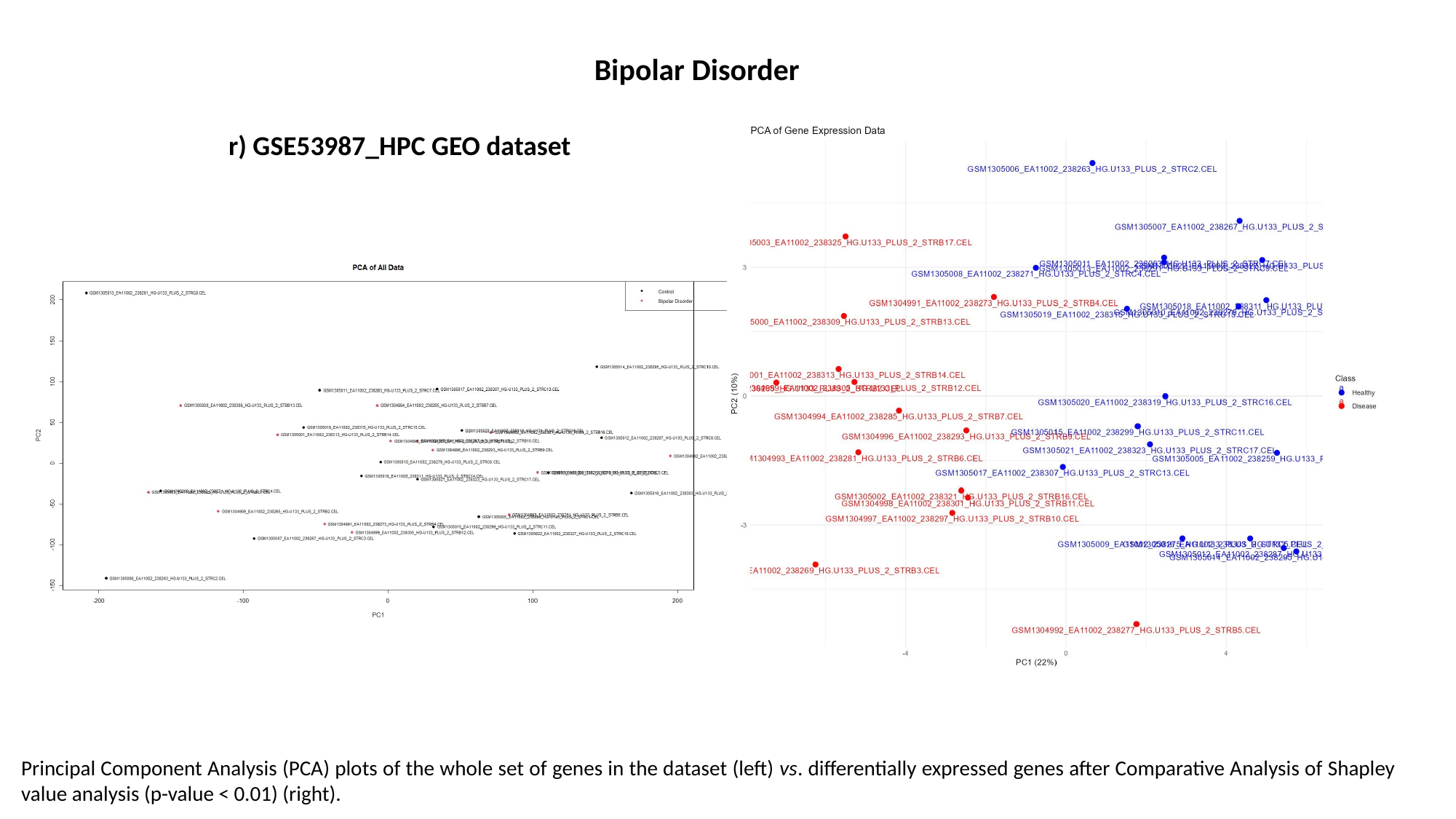

Bipolar Disorder
r) GSE53987_HPC GEO dataset
Principal Component Analysis (PCA) plots of the whole set of genes in the dataset (left) vs. differentially expressed genes after Comparative Analysis of Shapley value analysis (p-value < 0.01) (right).

## Slide 19
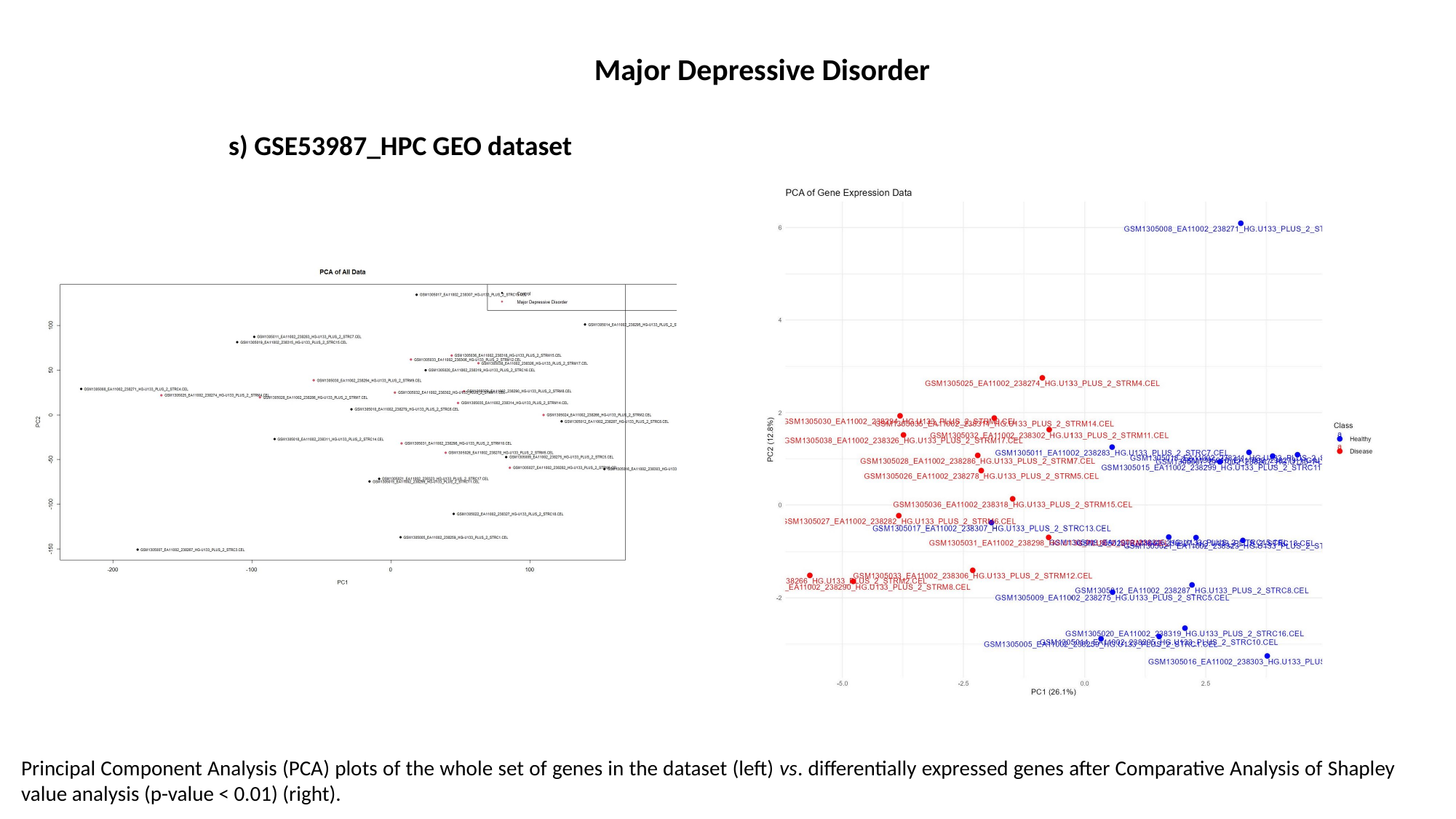

Major Depressive Disorder
s) GSE53987_HPC GEO dataset
Principal Component Analysis (PCA) plots of the whole set of genes in the dataset (left) vs. differentially expressed genes after Comparative Analysis of Shapley value analysis (p-value < 0.01) (right).
