# Supplementary material for: Enhancing Transcriptomic Insights into Neurological Disorders Through the Comparative Analysis of Shapley Values
Source: Curr Issues Mol Biol. 2024 Nov 29;46(12):13583–606. doi: 10.3390/cimb46120812 (PMC11726880; doi:10.3390/cimb46120812)
Supplement: Supplementary file 1 [file cimb-46-00812-s001.zip › SupplementaryFigure_S3.pptx]

## Slide 1
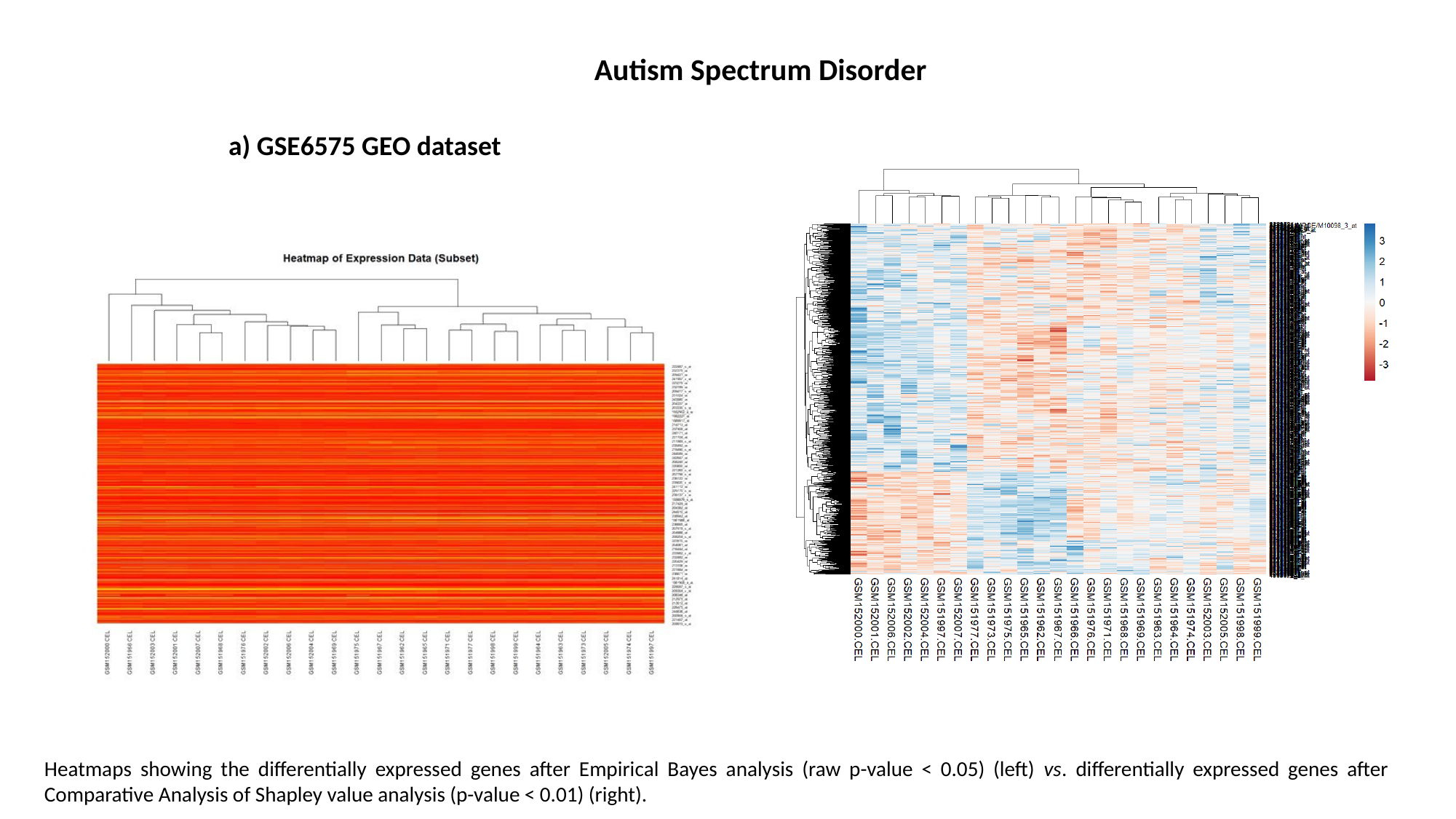

Autism Spectrum Disorder
a) GSE6575 GEO dataset
Heatmaps showing the differentially expressed genes after Empirical Bayes analysis (raw p-value < 0.05) (left) vs. differentially expressed genes after Comparative Analysis of Shapley value analysis (p-value < 0.01) (right).

## Slide 2
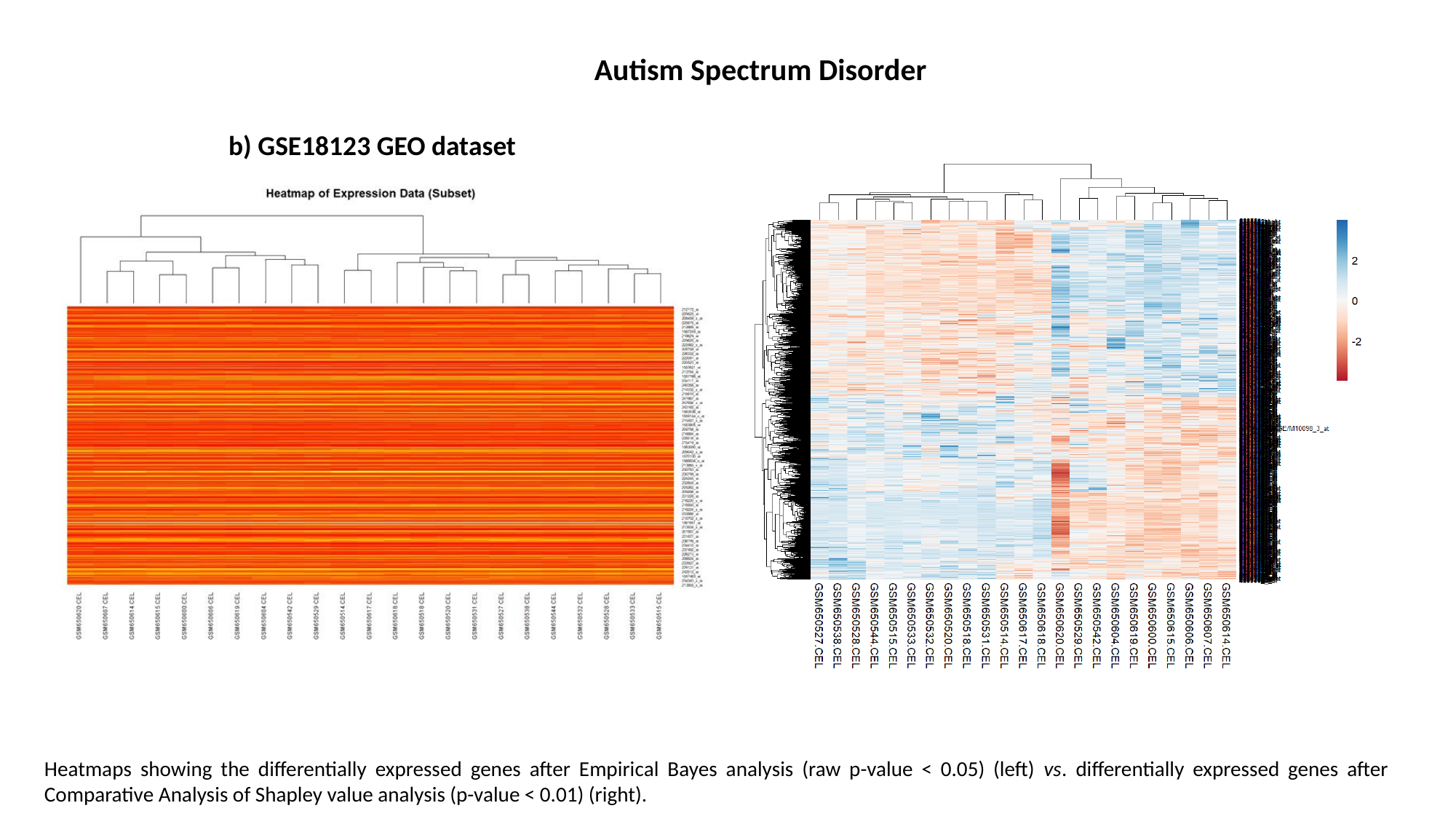

Autism Spectrum Disorder
b) GSE18123 GEO dataset
Heatmaps showing the differentially expressed genes after Empirical Bayes analysis (raw p-value < 0.05) (left) vs. differentially expressed genes after Comparative Analysis of Shapley value analysis (p-value < 0.01) (right).

## Slide 3
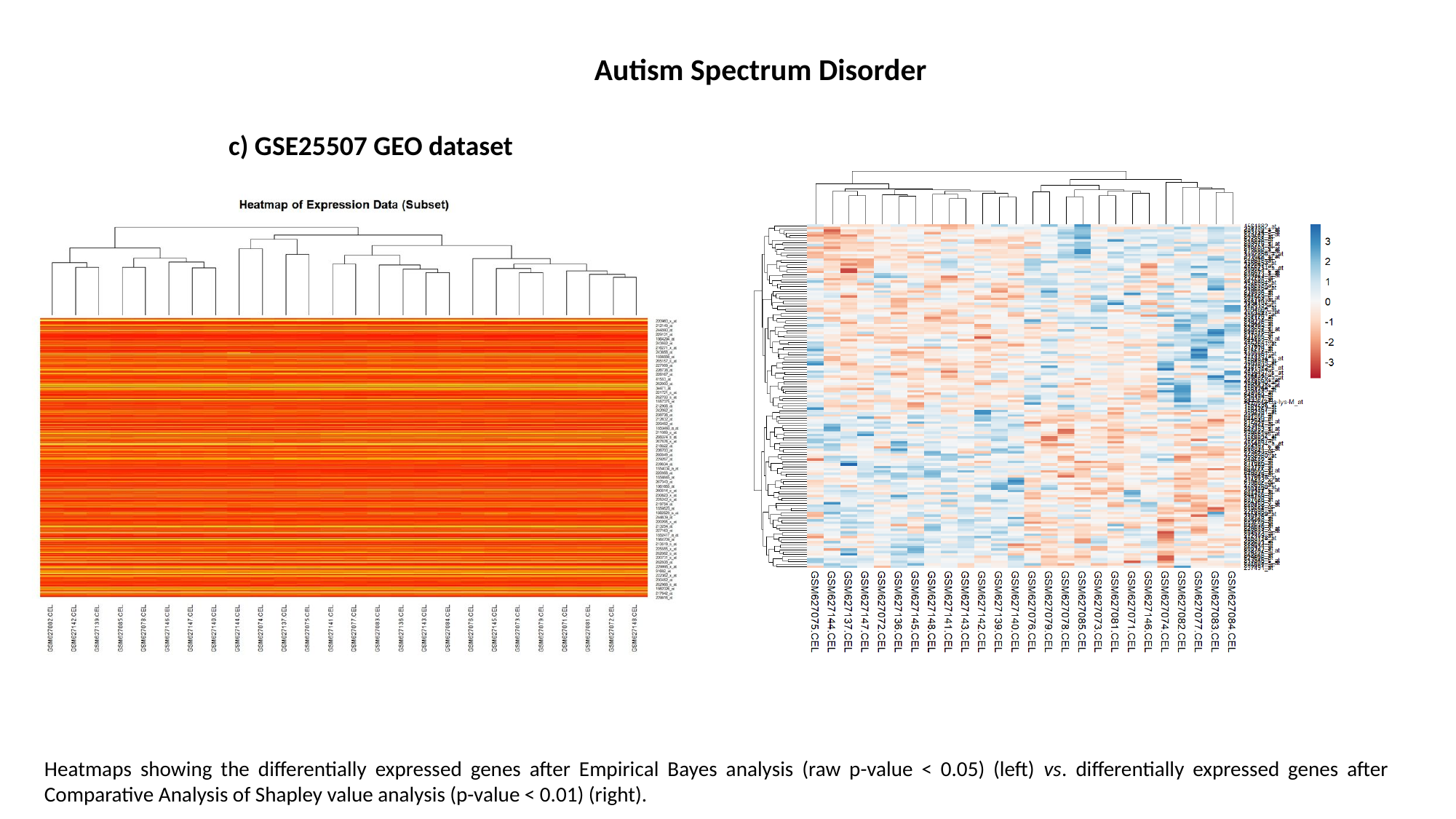

Autism Spectrum Disorder
c) GSE25507 GEO dataset
Heatmaps showing the differentially expressed genes after Empirical Bayes analysis (raw p-value < 0.05) (left) vs. differentially expressed genes after Comparative Analysis of Shapley value analysis (p-value < 0.01) (right).

## Slide 4
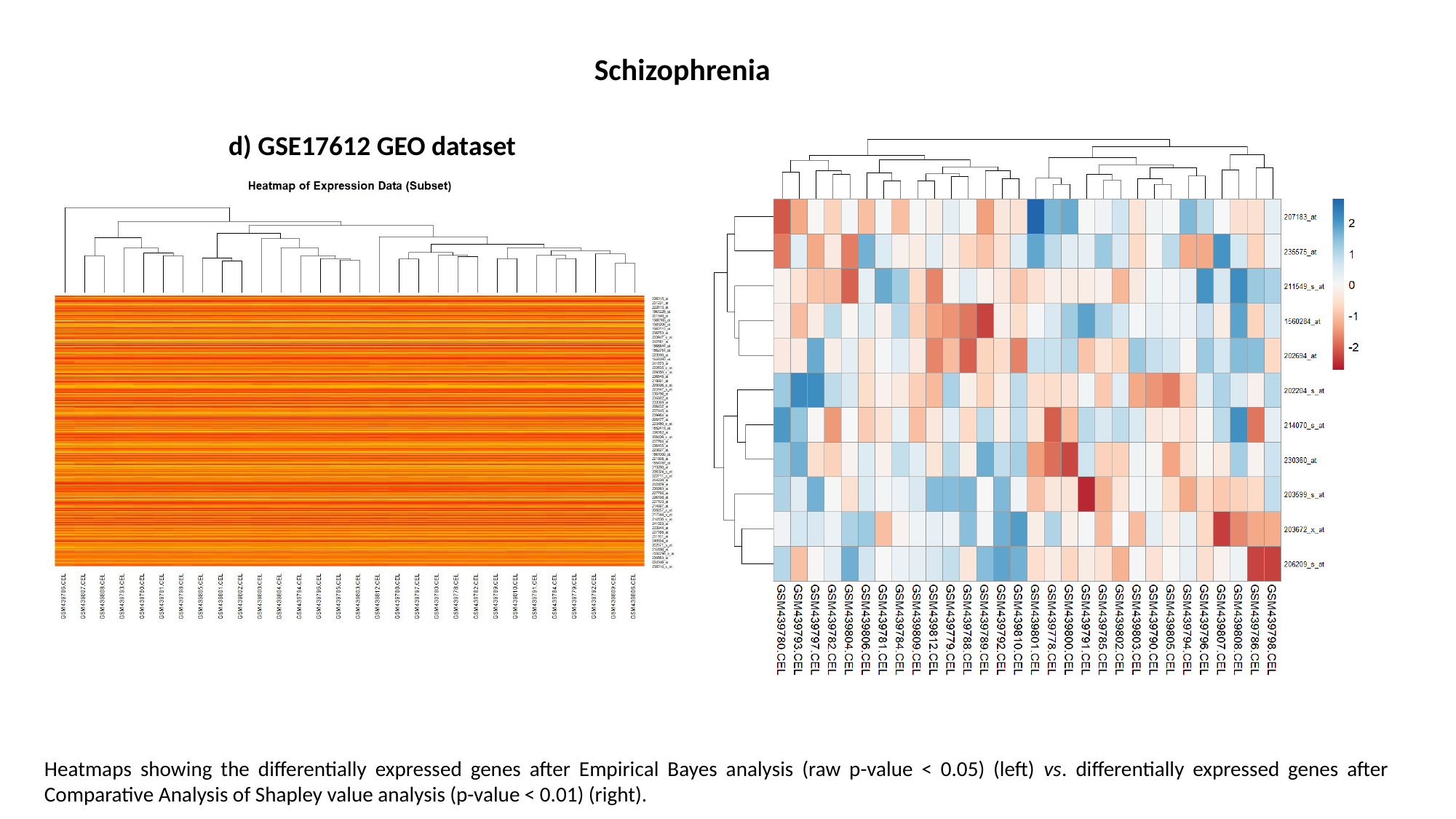

Schizophrenia
d) GSE17612 GEO dataset
Heatmaps showing the differentially expressed genes after Empirical Bayes analysis (raw p-value < 0.05) (left) vs. differentially expressed genes after Comparative Analysis of Shapley value analysis (p-value < 0.01) (right).

## Slide 5
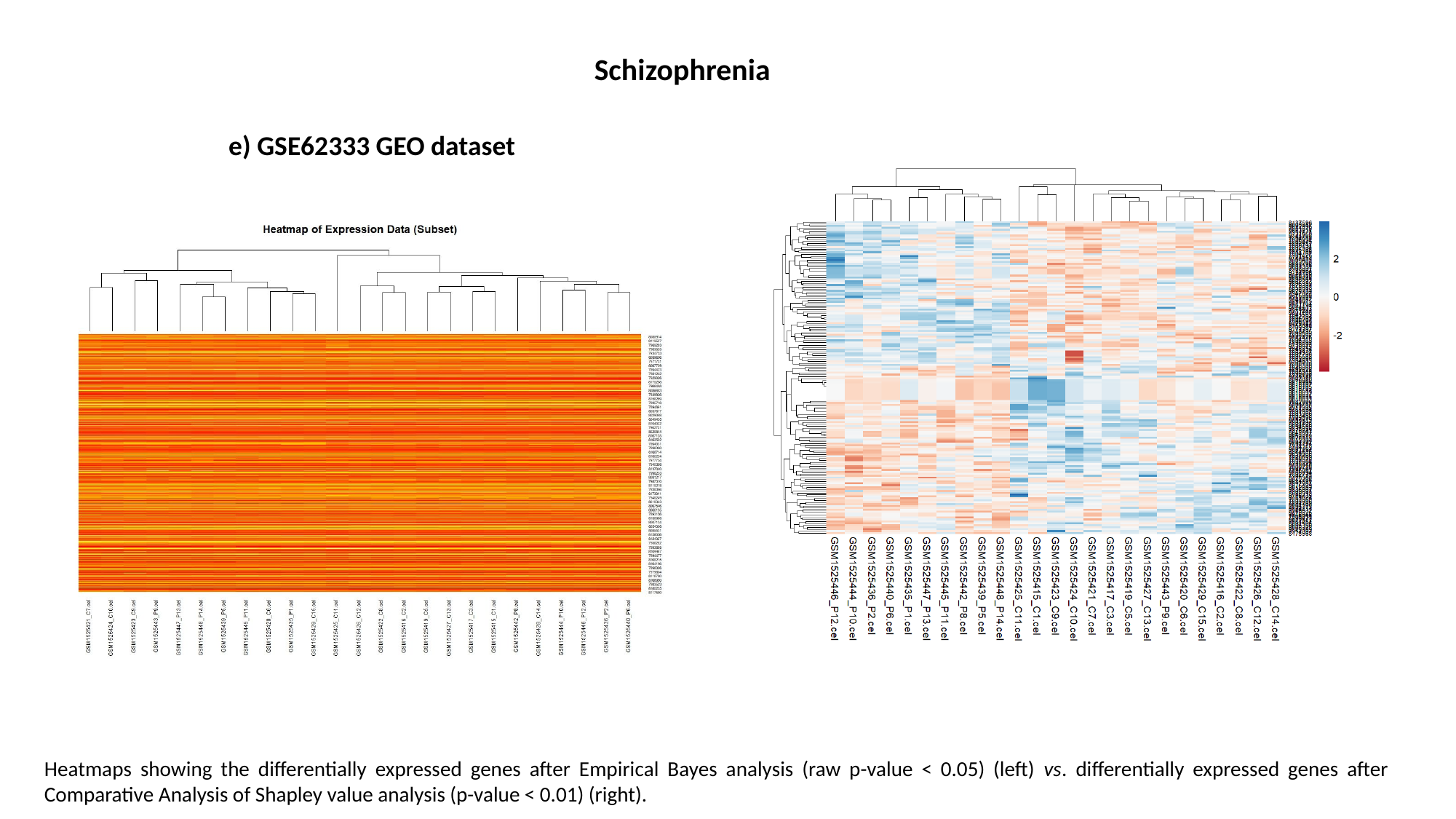

Schizophrenia
e) GSE62333 GEO dataset
Heatmaps showing the differentially expressed genes after Empirical Bayes analysis (raw p-value < 0.05) (left) vs. differentially expressed genes after Comparative Analysis of Shapley value analysis (p-value < 0.01) (right).

## Slide 6
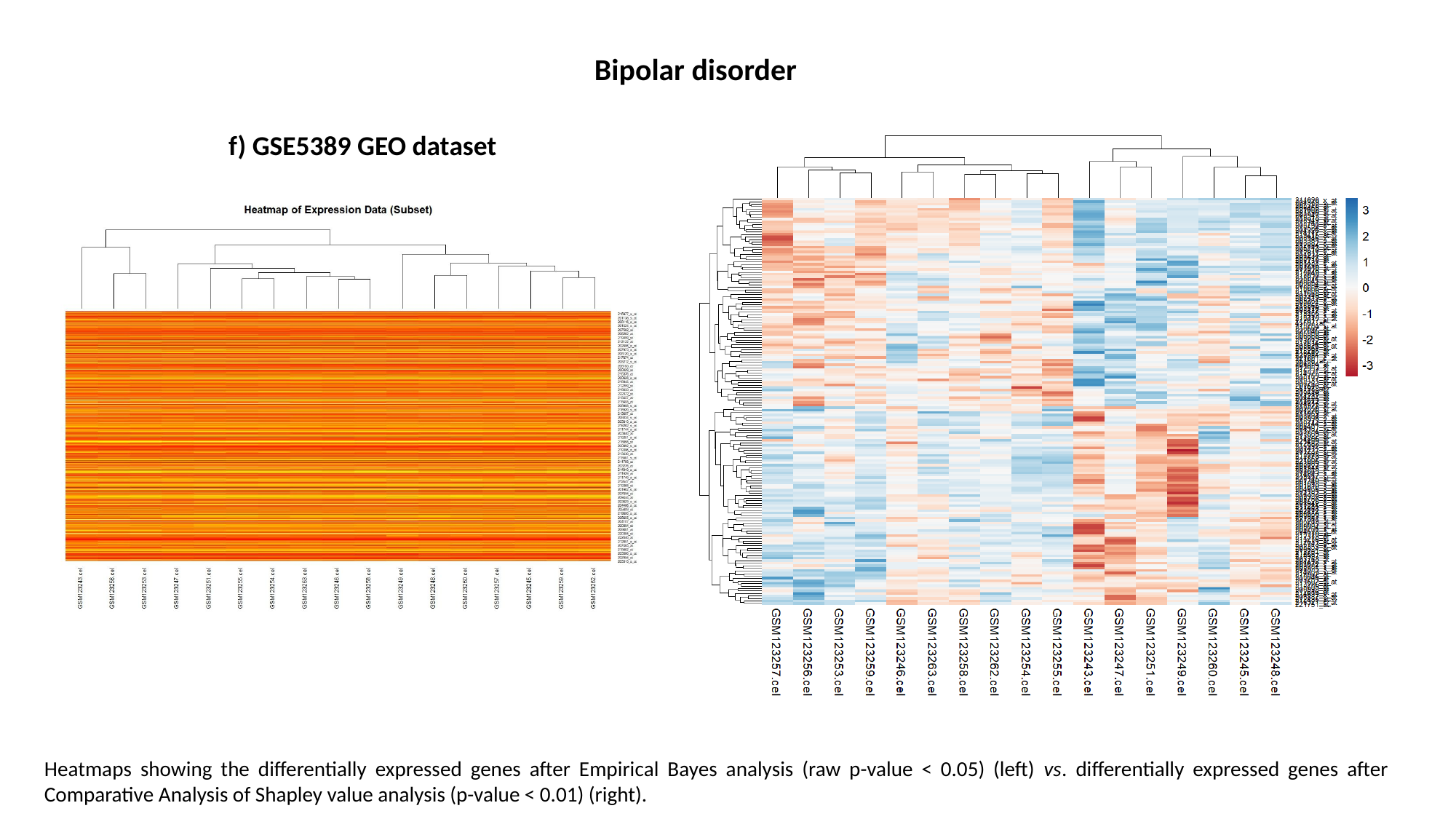

Bipolar disorder
f) GSE5389 GEO dataset
Heatmaps showing the differentially expressed genes after Empirical Bayes analysis (raw p-value < 0.05) (left) vs. differentially expressed genes after Comparative Analysis of Shapley value analysis (p-value < 0.01) (right).

## Slide 7
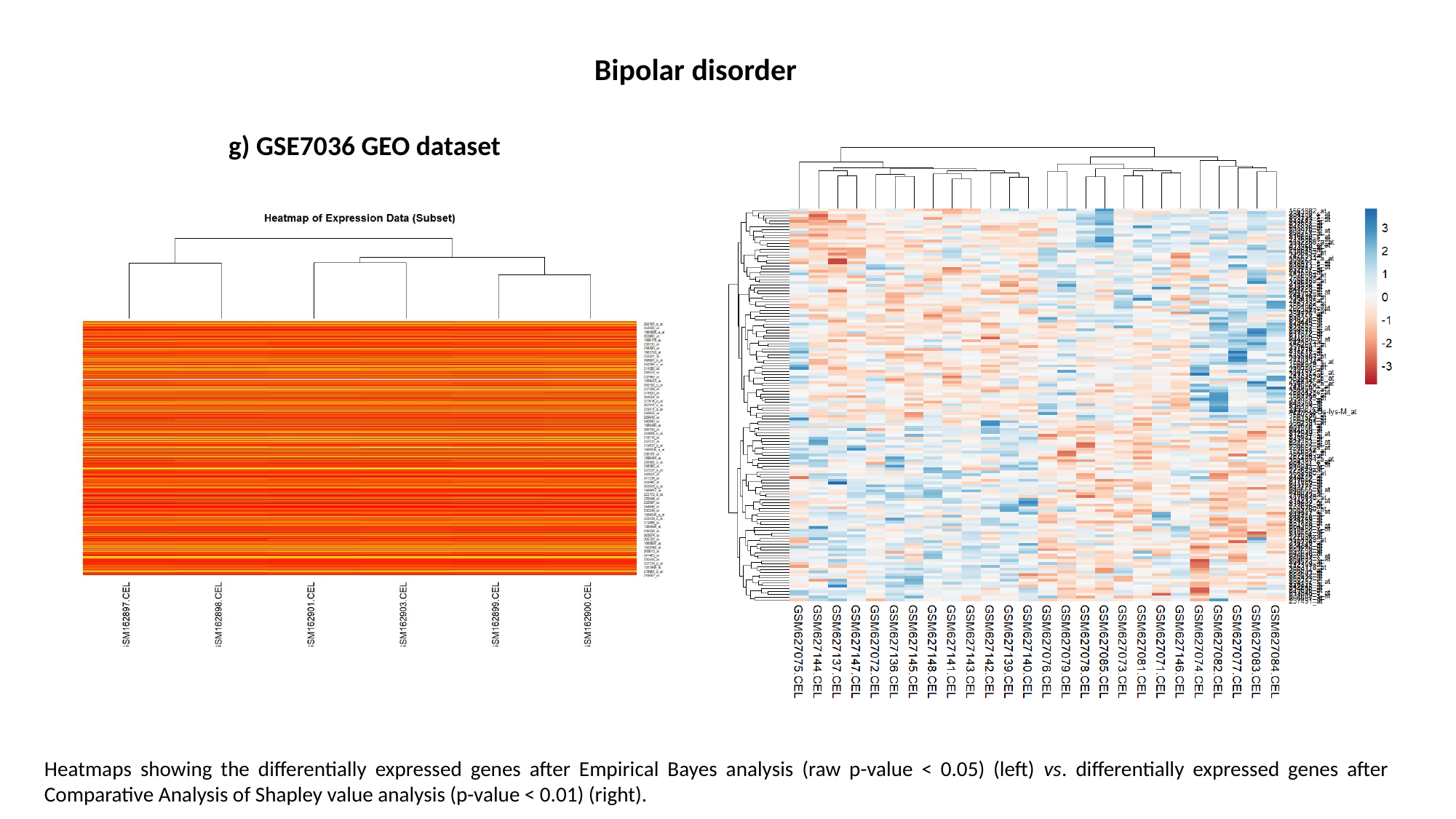

Bipolar disorder
g) GSE7036 GEO dataset
Heatmaps showing the differentially expressed genes after Empirical Bayes analysis (raw p-value < 0.05) (left) vs. differentially expressed genes after Comparative Analysis of Shapley value analysis (p-value < 0.01) (right).

## Slide 8
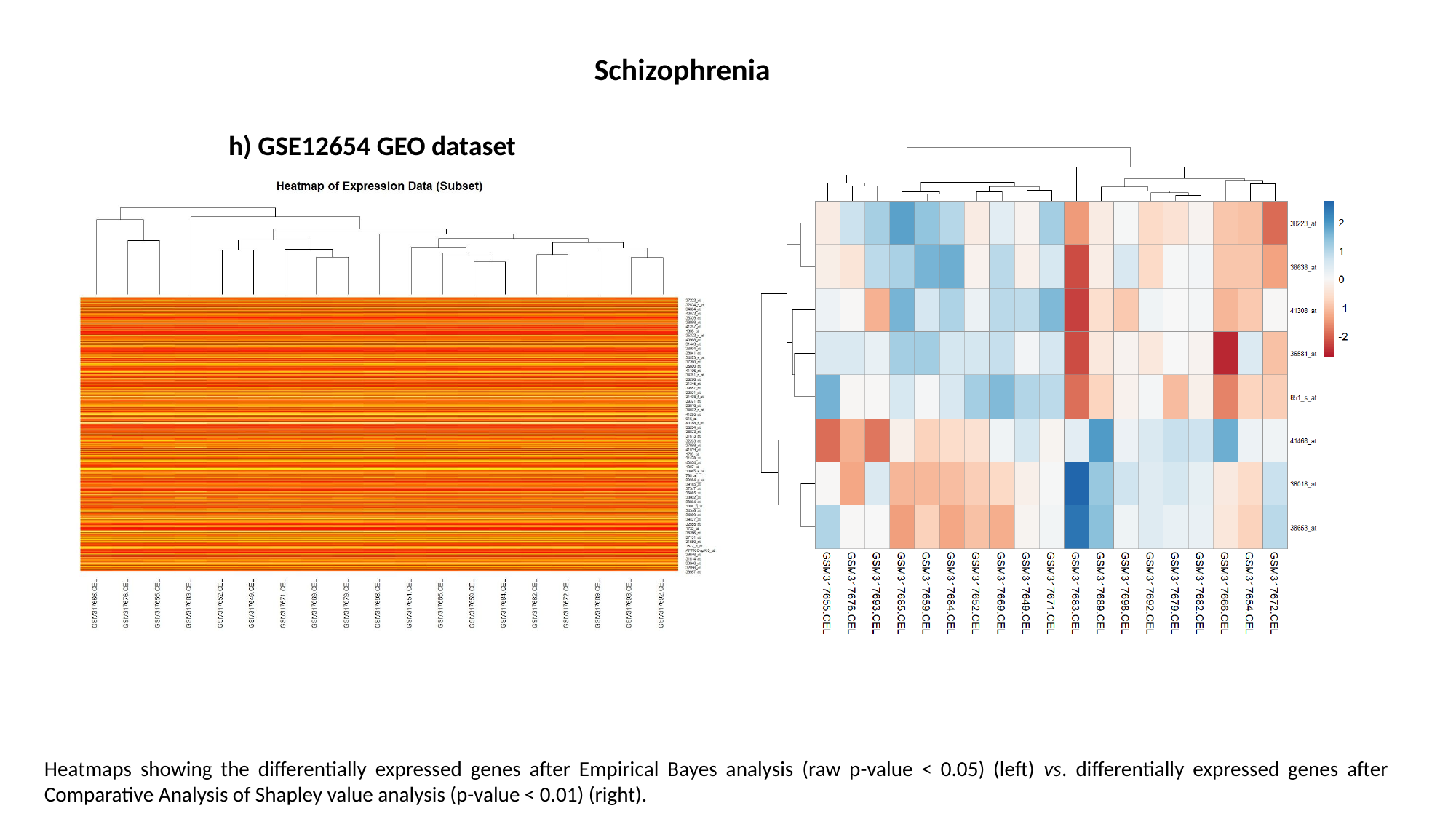

Schizophrenia
h) GSE12654 GEO dataset
Heatmaps showing the differentially expressed genes after Empirical Bayes analysis (raw p-value < 0.05) (left) vs. differentially expressed genes after Comparative Analysis of Shapley value analysis (p-value < 0.01) (right).

## Slide 9
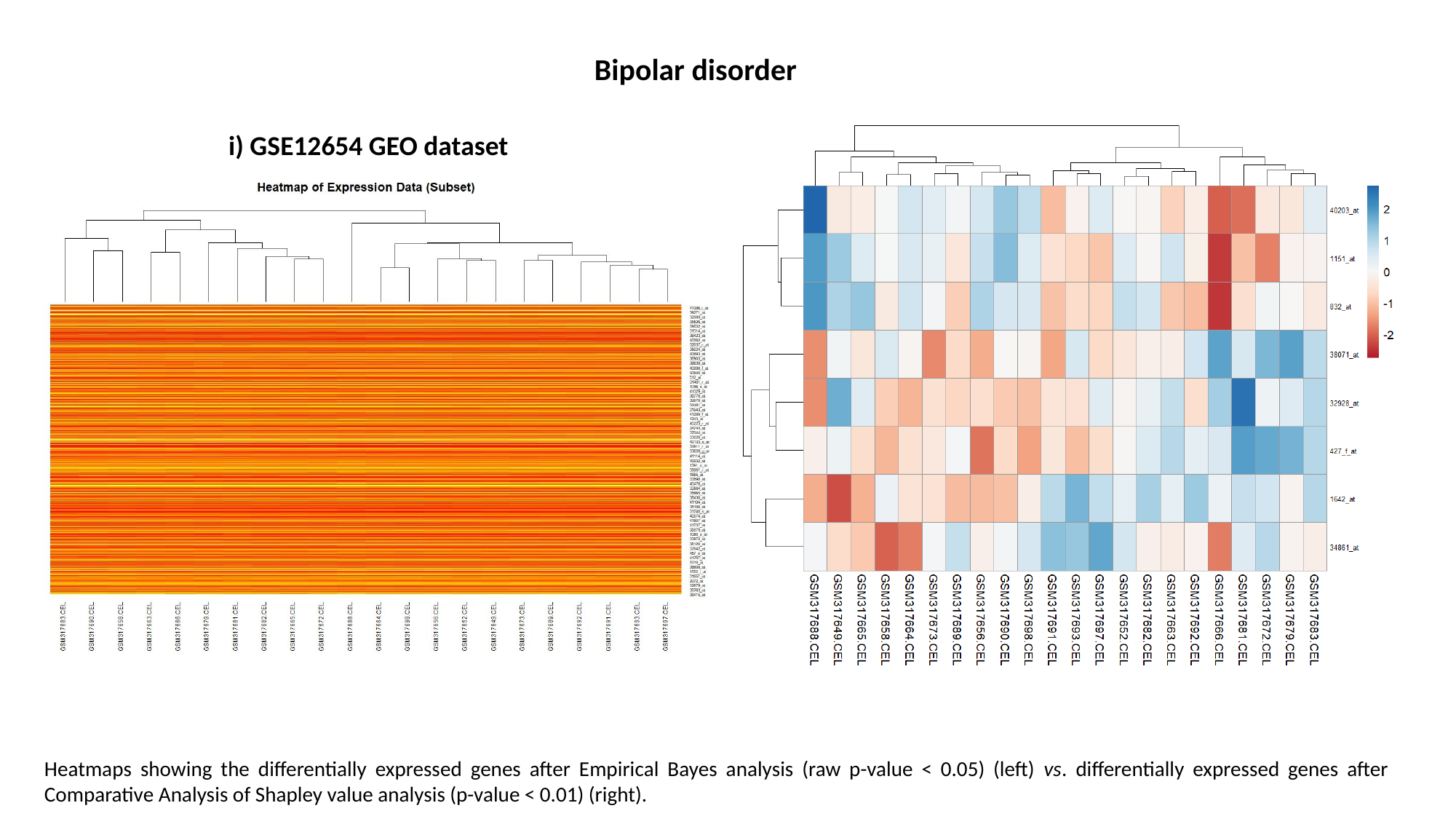

Bipolar disorder
i) GSE12654 GEO dataset
Heatmaps showing the differentially expressed genes after Empirical Bayes analysis (raw p-value < 0.05) (left) vs. differentially expressed genes after Comparative Analysis of Shapley value analysis (p-value < 0.01) (right).

## Slide 10
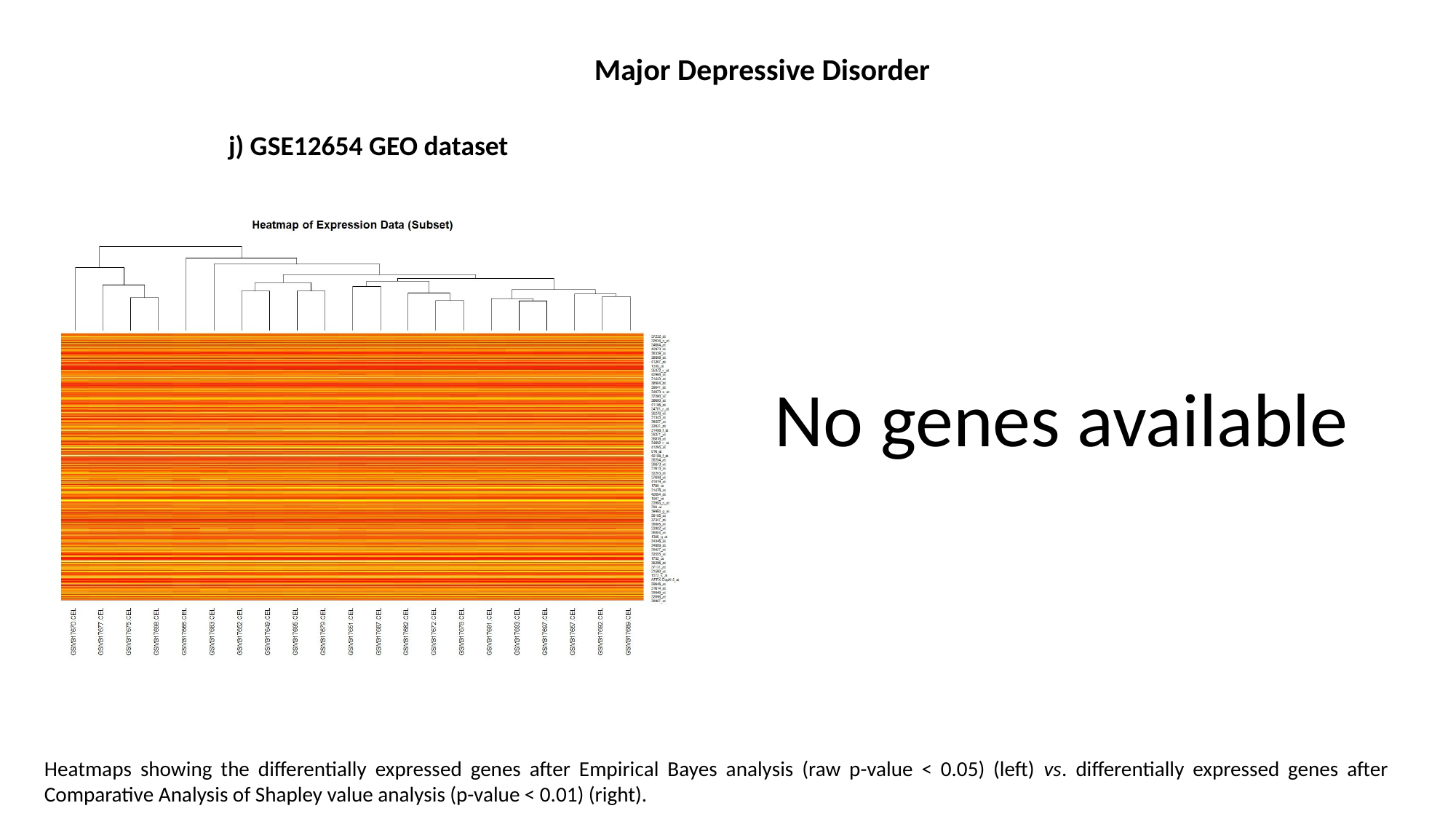

Major Depressive Disorder
j) GSE12654 GEO dataset
No genes available
Heatmaps showing the differentially expressed genes after Empirical Bayes analysis (raw p-value < 0.05) (left) vs. differentially expressed genes after Comparative Analysis of Shapley value analysis (p-value < 0.01) (right).

## Slide 11
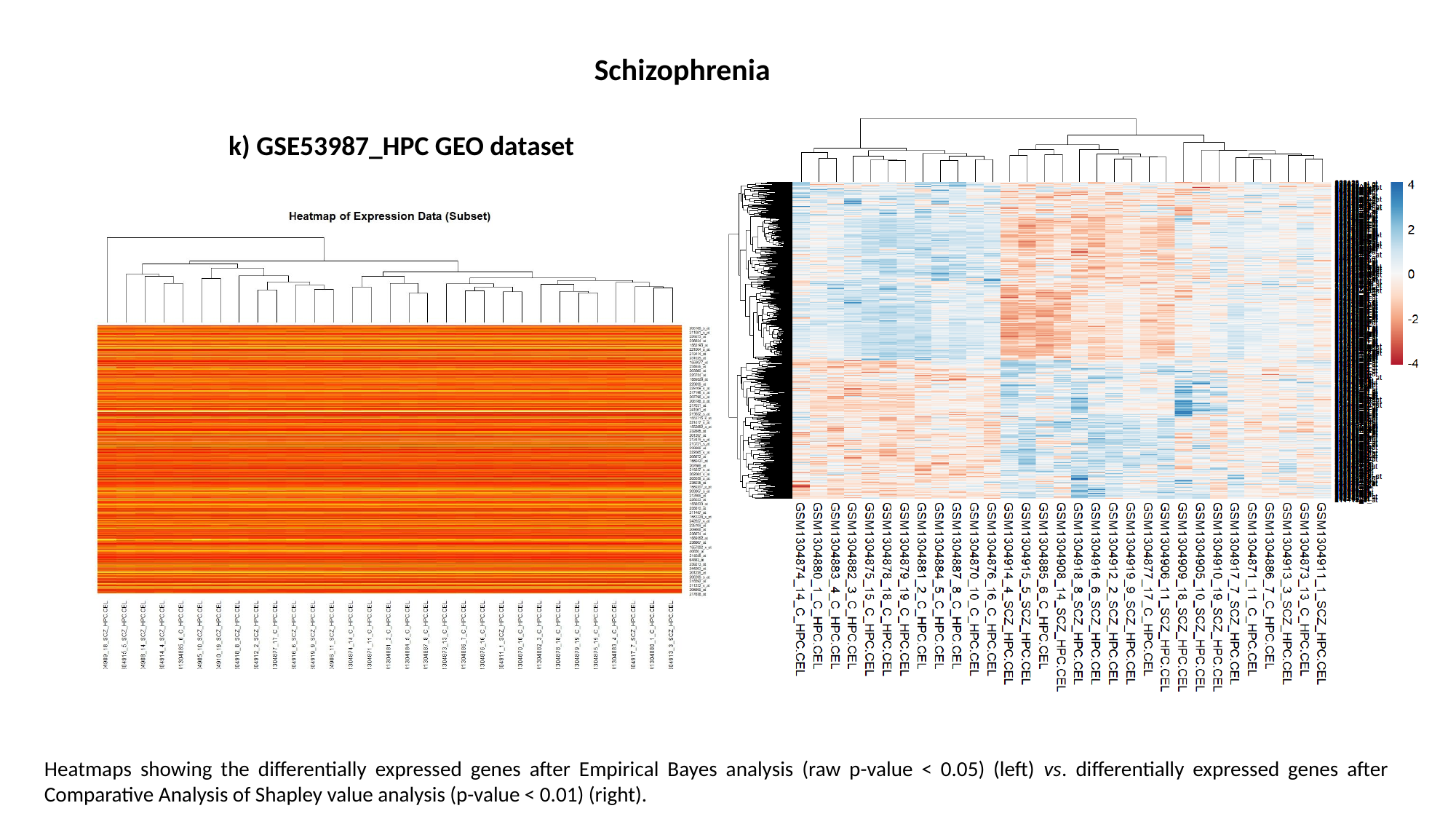

Schizophrenia
k) GSE53987_HPC GEO dataset
Heatmaps showing the differentially expressed genes after Empirical Bayes analysis (raw p-value < 0.05) (left) vs. differentially expressed genes after Comparative Analysis of Shapley value analysis (p-value < 0.01) (right).

## Slide 12
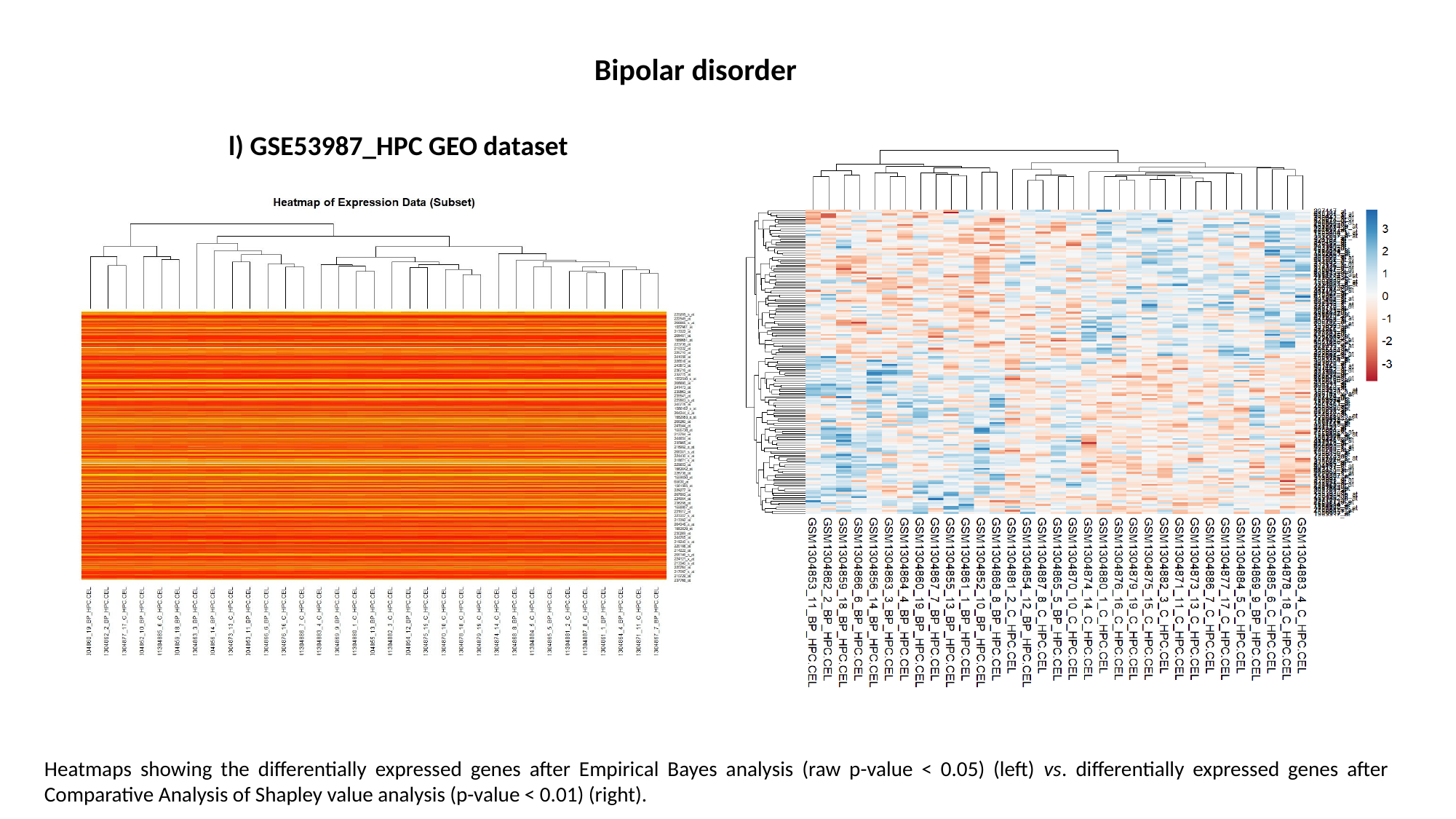

Bipolar disorder
l) GSE53987_HPC GEO dataset
Heatmaps showing the differentially expressed genes after Empirical Bayes analysis (raw p-value < 0.05) (left) vs. differentially expressed genes after Comparative Analysis of Shapley value analysis (p-value < 0.01) (right).

## Slide 13
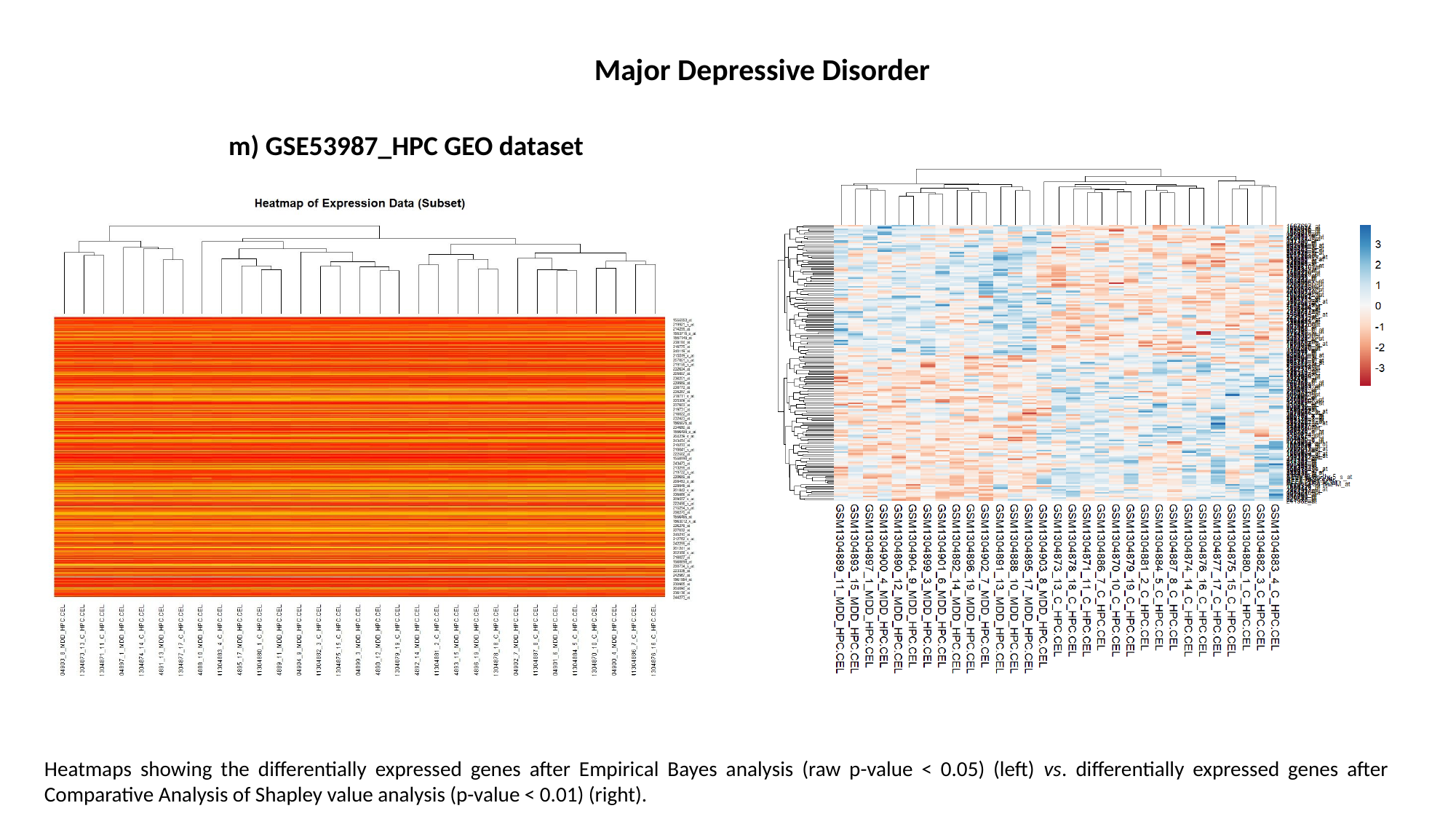

Major Depressive Disorder
m) GSE53987_HPC GEO dataset
Heatmaps showing the differentially expressed genes after Empirical Bayes analysis (raw p-value < 0.05) (left) vs. differentially expressed genes after Comparative Analysis of Shapley value analysis (p-value < 0.01) (right).

## Slide 14
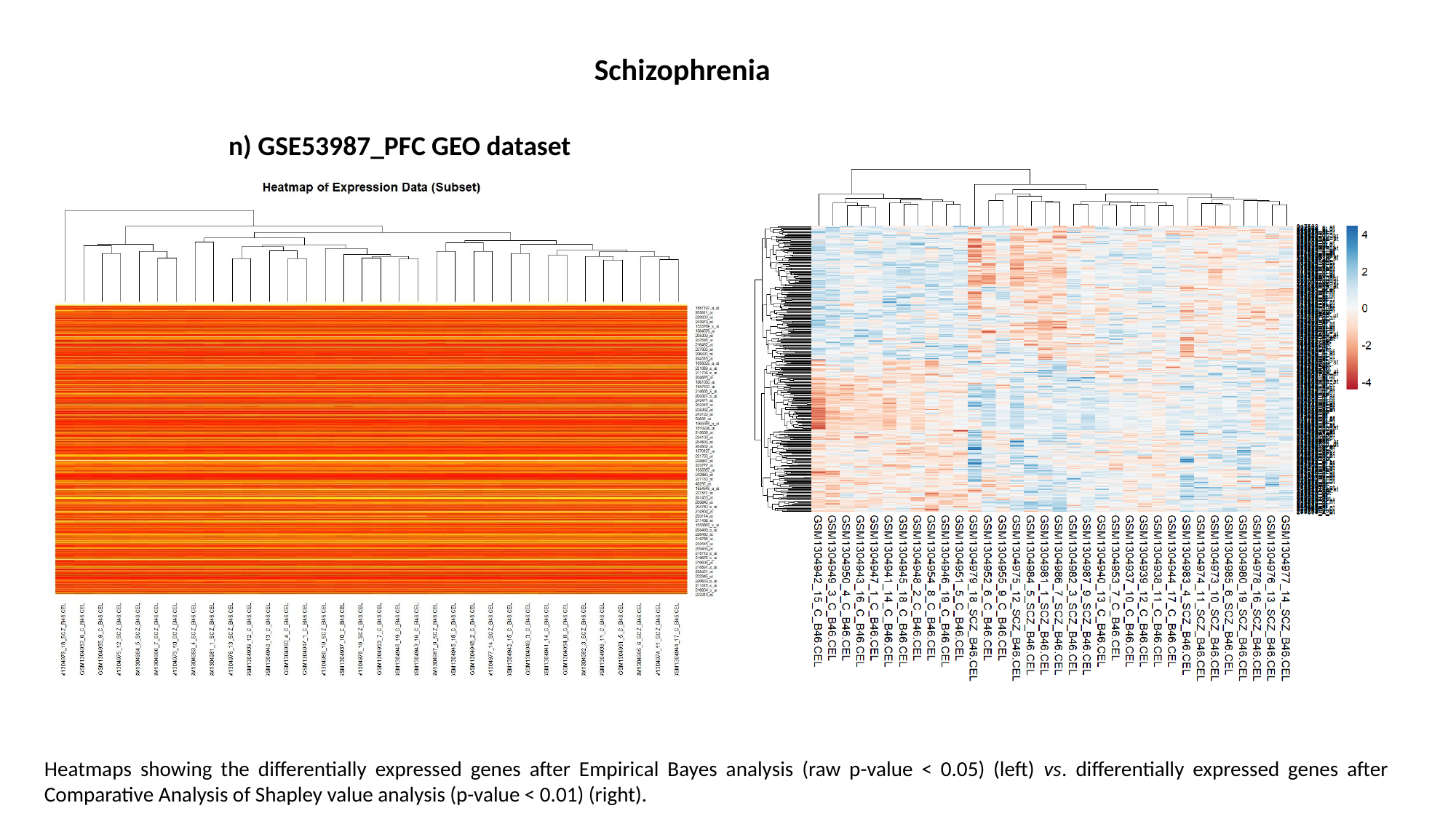

Schizophrenia
n) GSE53987_PFC GEO dataset
Heatmaps showing the differentially expressed genes after Empirical Bayes analysis (raw p-value < 0.05) (left) vs. differentially expressed genes after Comparative Analysis of Shapley value analysis (p-value < 0.01) (right).

## Slide 15
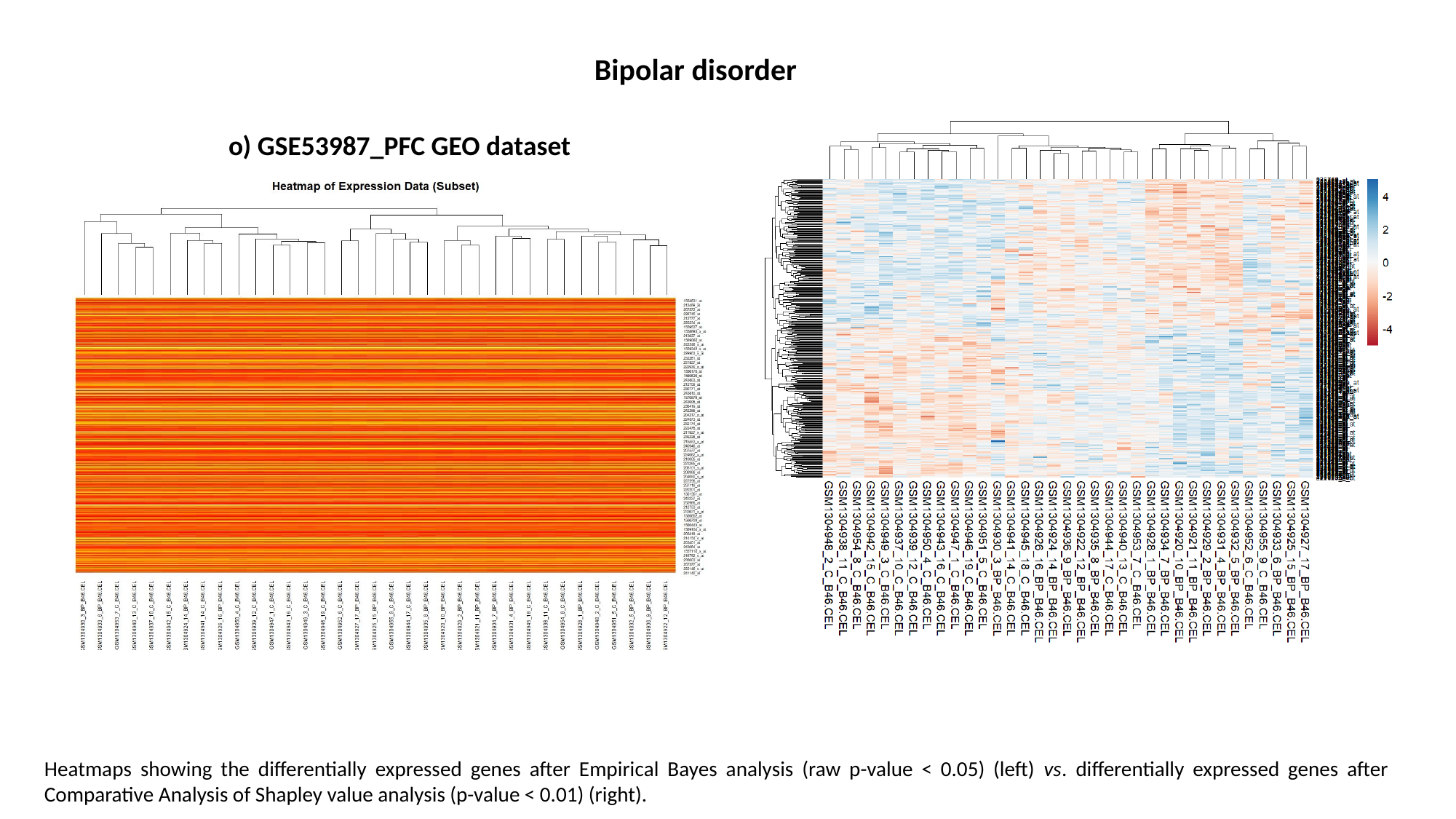

Bipolar disorder
o) GSE53987_PFC GEO dataset
Heatmaps showing the differentially expressed genes after Empirical Bayes analysis (raw p-value < 0.05) (left) vs. differentially expressed genes after Comparative Analysis of Shapley value analysis (p-value < 0.01) (right).

## Slide 16
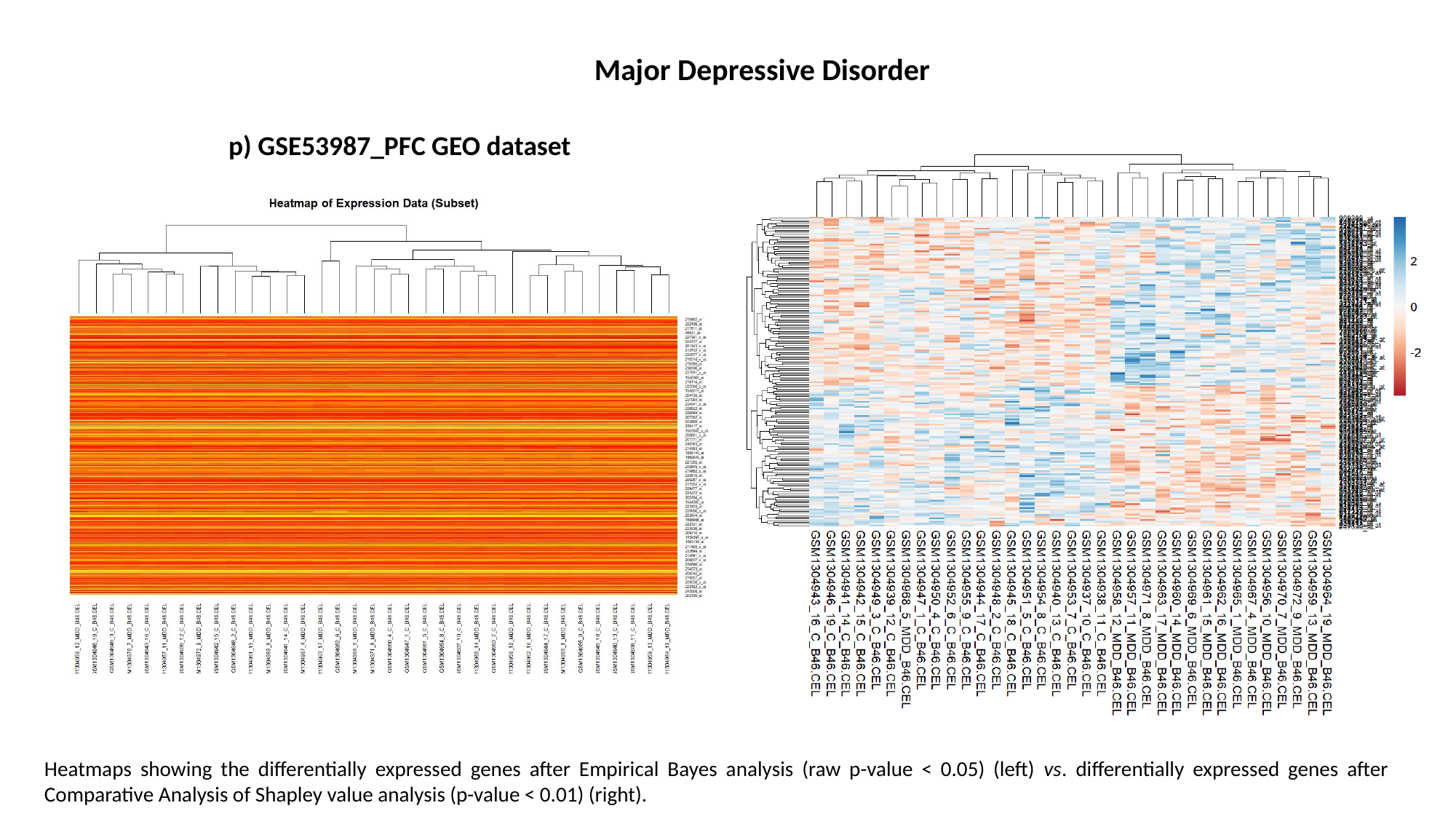

Major Depressive Disorder
p) GSE53987_PFC GEO dataset
Heatmaps showing the differentially expressed genes after Empirical Bayes analysis (raw p-value < 0.05) (left) vs. differentially expressed genes after Comparative Analysis of Shapley value analysis (p-value < 0.01) (right).

## Slide 17
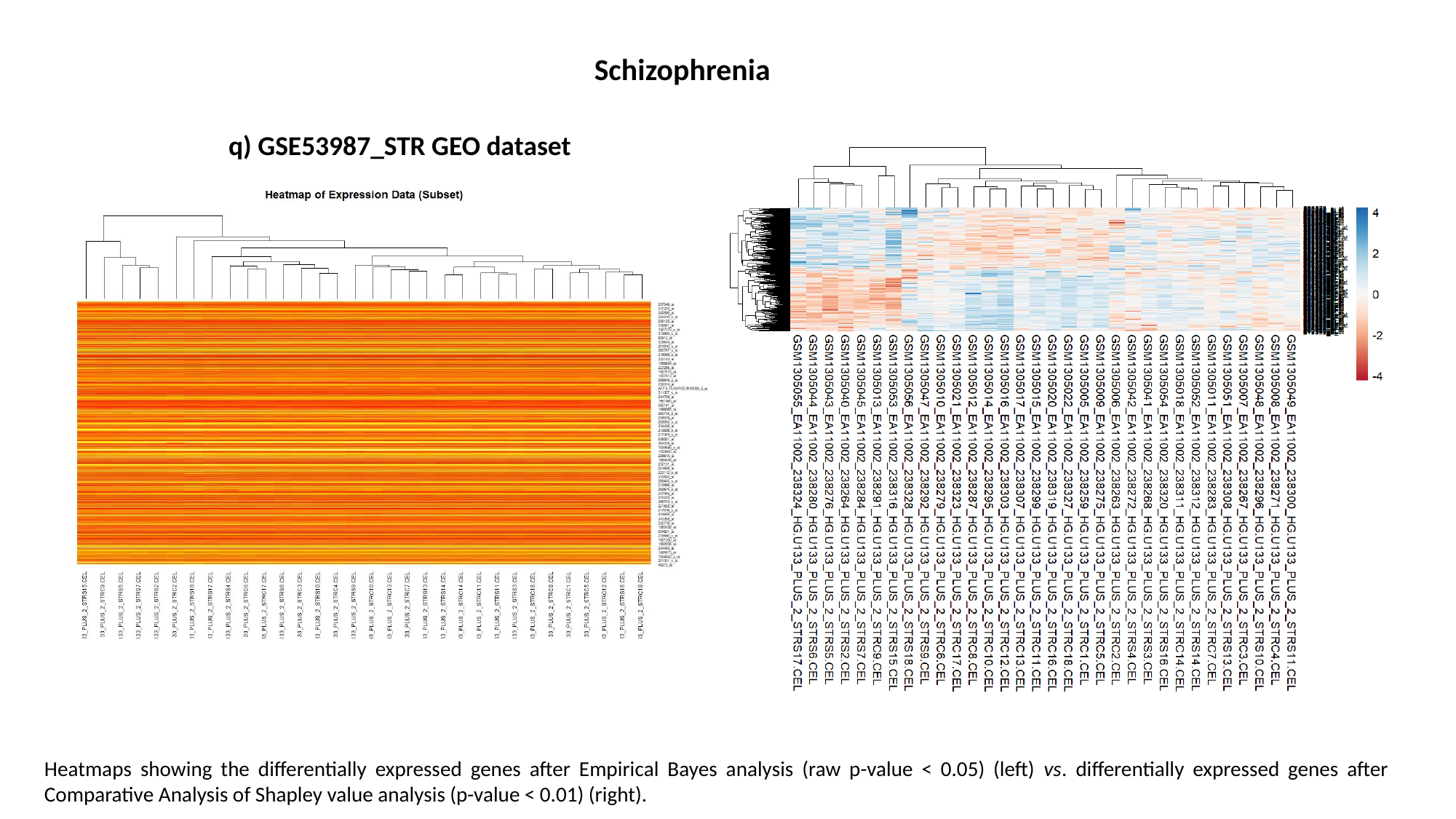

Schizophrenia
q) GSE53987_STR GEO dataset
Heatmaps showing the differentially expressed genes after Empirical Bayes analysis (raw p-value < 0.05) (left) vs. differentially expressed genes after Comparative Analysis of Shapley value analysis (p-value < 0.01) (right).

## Slide 18
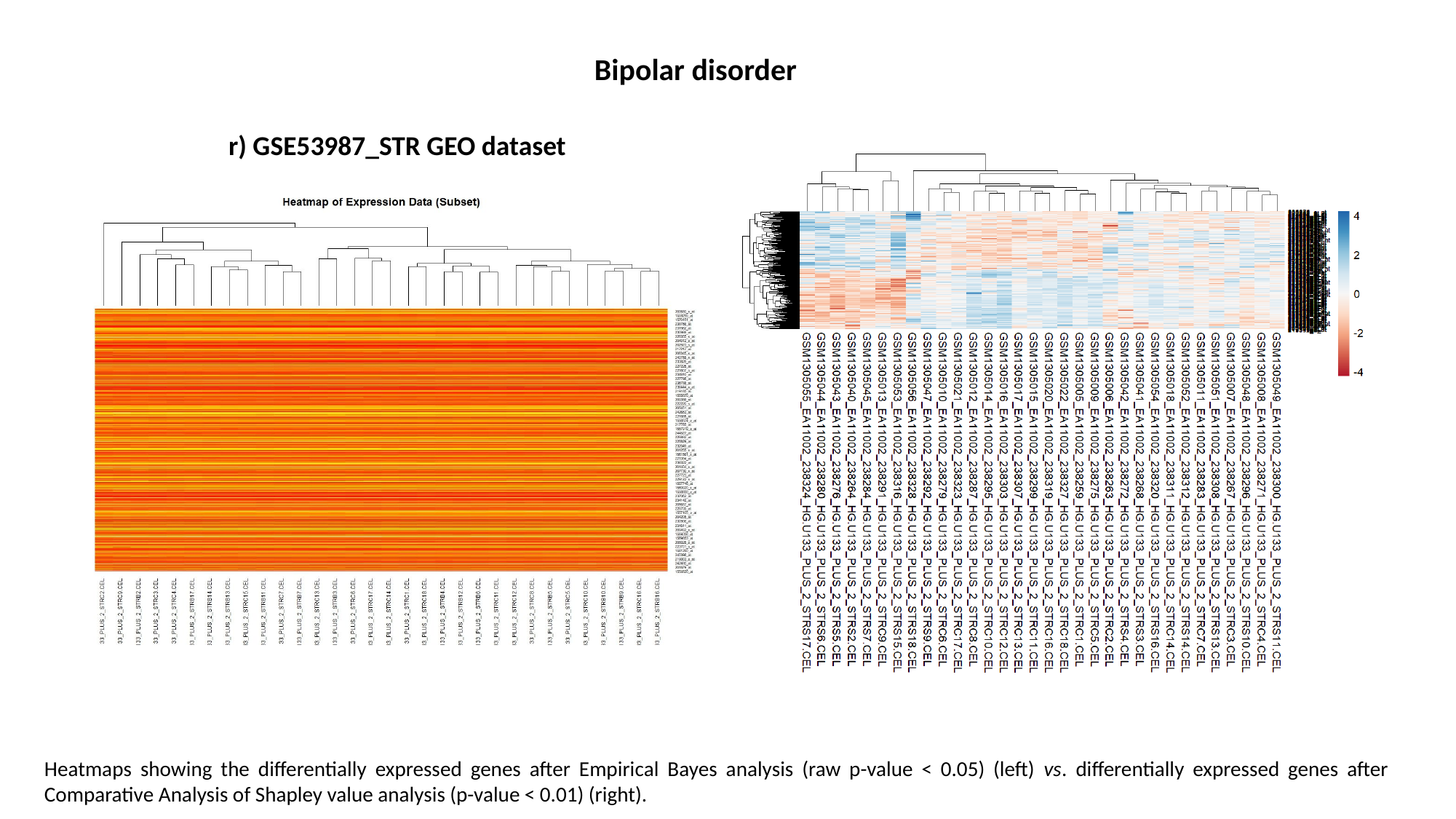

Bipolar disorder
r) GSE53987_STR GEO dataset
Heatmaps showing the differentially expressed genes after Empirical Bayes analysis (raw p-value < 0.05) (left) vs. differentially expressed genes after Comparative Analysis of Shapley value analysis (p-value < 0.01) (right).

## Slide 19
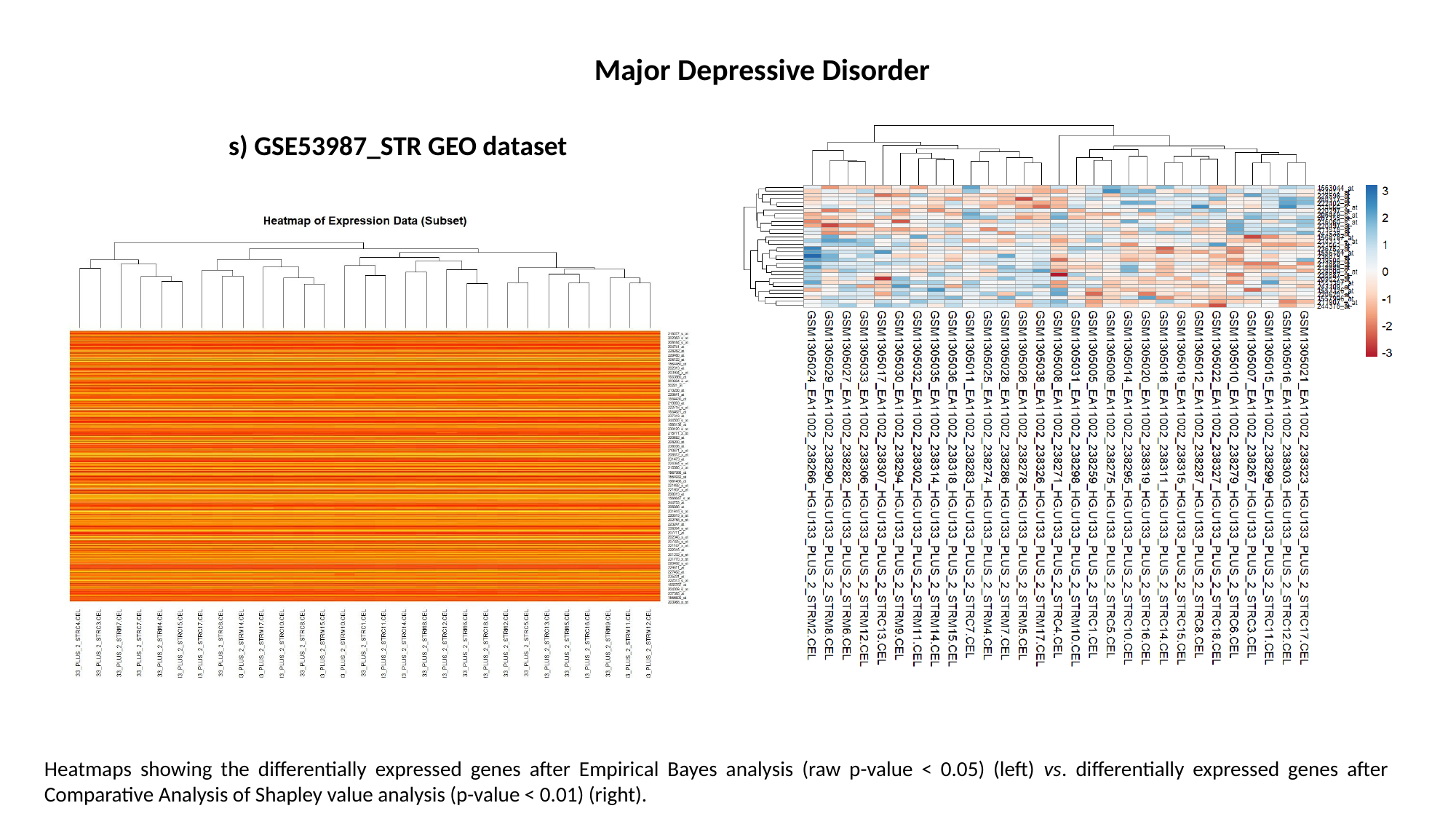

Major Depressive Disorder
s) GSE53987_STR GEO dataset
Heatmaps showing the differentially expressed genes after Empirical Bayes analysis (raw p-value < 0.05) (left) vs. differentially expressed genes after Comparative Analysis of Shapley value analysis (p-value < 0.01) (right).
